# Detecting purely epistatic multi-locus interactions by an omnibus permutation test on ensembles of two-locus analyses

**DOI:** 10.1186/1471-2105-10-294

**Published:** 2009-09-17

**Authors:** Waranyu Wongseree, Anunchai Assawamakin, Theera Piroonratana, Saravudh Sinsomros, Chanin Limwongse, Nachol Chaiyaratana

**Affiliations:** 1 Department of Electrical Engineering, Faculty of Engineering, King Mongkut's University of Technology North Bangkok, 1518 Piboolsongkram Road, Bangsue, Bangkok 10800, Thailand; 2 Division of Molecular Genetics, Department of Research and Development, Faculty of Medicine Siriraj Hospital, Mahidol University, 2 Prannok Road, Bangkoknoi, Bangkok 10700, Thailand

## Abstract

**Background:**

Purely epistatic multi-locus interactions cannot generally be detected via single-locus analysis in case-control studies of complex diseases. Recently, many two-locus and multi-locus analysis techniques have been shown to be promising for the epistasis detection. However, exhaustive multi-locus analysis requires prohibitively large computational efforts when problems involve large-scale or genome-wide data. Furthermore, there is no explicit proof that a combination of multiple two-locus analyses can lead to the correct identification of multi-locus interactions.

**Results:**

The proposed 2LOmb algorithm performs an omnibus permutation test on ensembles of two-locus analyses. The algorithm consists of four main steps: two-locus analysis, a permutation test, global *p*-value determination and a progressive search for the best ensemble. 2LOmb is benchmarked against an exhaustive two-locus analysis technique, a set association approach, a correlation-based feature selection (CFS) technique and a tuned ReliefF (TuRF) technique. The simulation results indicate that 2LOmb produces a low false-positive error. Moreover, 2LOmb has the best performance in terms of an ability to identify all causative single nucleotide polymorphisms (SNPs) and a low number of output SNPs in purely epistatic two-, three- and four-locus interaction problems. The interaction models constructed from the 2LOmb outputs via a multifactor dimensionality reduction (MDR) method are also included for the confirmation of epistasis detection. 2LOmb is subsequently applied to a type 2 diabetes mellitus (T2D) data set, which is obtained as a part of the UK genome-wide genetic epidemiology study by the Wellcome Trust Case Control Consortium (WTCCC). After primarily screening for SNPs that locate within or near 372 candidate genes and exhibit no marginal single-locus effects, the T2D data set is reduced to 7,065 SNPs from 370 genes. The 2LOmb search in the reduced T2D data reveals that four intronic SNPs in *PGM1 *(phosphoglucomutase 1), two intronic SNPs in *LMX1A *(LIM homeobox transcription factor 1, alpha), two intronic SNPs in *PARK2 *(Parkinson disease (autosomal recessive, juvenile) 2, parkin) and three intronic SNPs in *GYS2 *(glycogen synthase 2 (liver)) are associated with the disease. The 2LOmb result suggests that there is no interaction between each pair of the identified genes that can be described by purely epistatic two-locus interaction models. Moreover, there are no interactions between these four genes that can be described by purely epistatic multi-locus interaction models with marginal two-locus effects. The findings provide an alternative explanation for the aetiology of T2D in a UK population.

**Conclusion:**

An omnibus permutation test on ensembles of two-locus analyses can detect purely epistatic multi-locus interactions with marginal two-locus effects. The study also reveals that SNPs from large-scale or genome-wide case-control data which are discarded after single-locus analysis detects no association can still be useful for genetic epidemiology studies.

## Background

Complex diseases cannot generally be explained by Mendelian inheritance [1] because they are influenced by gene-gene and gene-environment interactions. Many common diseases such as asthma, cancer, diabetes, hypertension and obesity are widely accepted and acknowledged to be results of complex interactions between multiple genetic factors [2]. Attempts to identify factors that could be the causes of complex diseases have led to many genome-wide association studies [3,4]. Raw results from these attempts produce a large amount of single nucleotide polymorphism (SNP) data from every individual participating in the trials.

For genetic epidemiologists, data sets from genome-wide association studies present many challenges, particularly the correct identification of SNPs that associate with the disease of interest from all available SNPs [5]. This challenge can be treated as a pattern recognition problem which aims to identify an attribute or SNP set that can lead to the correct classification of recruited samples. Heidema et al. [5] and Motsinger et al. [6] have reviewed and identified many machine learning techniques that are suitable to the task. Among many strategies and techniques, the protocol that appears to be most promising for genome-wide association studies involves two main steps: SNP set reduction and classification model construction [7]. From a machine learning viewpoint, attribute selection techniques can be divided into three main categories: filter, wrapper and embedded approaches [8]. In a filter approach, a measure or an index is used to determine the correlation between attributes and classes, e.g. affected and unaffected status in a case-control study. Attributes that are deemed to be important for the classification according to the measure are then selected. The filter approach includes *χ*^2 ^and odds ratio tests [9,10], omnibus permutation tests [11-13], a correlation-based feature selection technique [14], a ReliefF technique [15] and a tuned ReliefF technique [16]. In a wrapper approach, the significance of an attribute subset is evaluated from the classification performance by a classifier. The capability of the wrapper approach to identify significant attributes thus depends on the chosen classifier and the search algorithm for the identification of the best attribute subset. Combinatorial [17] and restricted partitioning methods [18], a multifactor dimensionality reduction method [19-25] and a polymorphism interaction analysis technique [26] are examples of the wrapper approach. An embedded approach concentrates on informative attributes during the construction of a classification model. Examples of the embedded approach include a genetic algorithm with Boolean algebra [27], genetic programming based decision trees [28,29], random forests [30-32] and evolutionary neural networks [33,34]. Based on this categorisation, classification models are not direct outputs from filter-based techniques. On the other hand, classification models are readily prepared as outputs from the wrapper and embedded approaches. In other words, the last two approaches can also be regarded as classification model construction techniques.

The success of the two-step pattern recognition approach relies heavily on the attribute selection step [14]. In case-control studies, epistatic effects play a vital role in establishing the difficulty level of SNP screening problems [35,36]. Epistasis in the simplest form can be represented by disease models that require genotype inputs from two interacting SNPs [37,38]. Many attempts have been made to produce consistent definitions and categorisation of different types of epistasis models [2,35,39-41]. According to Musani et al. [2], a pure epistasis model [42] is difficult because each SNP exhibits no marginal single-locus effect in the model. As a result, it is impossible to detect the pure epistasis by univariate statistical tests. Examples of complex diseases that case-control studies have uncovered putatively pure epistasis include type 2 diabetes mellitus (T2D) [43-46] and metabolic syndrome [47]. Due to the difficulty of screening for each SNP independently, it is suggested that attention should be focused on the analysis of differences between two-locus genotype distribution within case and control groups [40] and multi-locus Bayesian statistical analysis [48,49].

A number of SNP screening and association detection techniques have adopted the two-locus genotype monitoring strategy as their core engines [40,50-52]. The search for interactions can be carried out via either exhaustive analysis [52] or the analysis that can be divided into two stages, incorporating single-locus analysis for the pre-screening purpose [40,50,51]. In the two-stage mode, at least one SNP that involves in the construction of two-locus genotype unit must be a strong candidate for the association explanation, usually verified through univariate statistical tests. Each mode of the two-locus analysis possesses different strengths and weaknesses. The exhaustive analysis has a full capability of detecting pure epistasis but requires larger computational efforts [52]. In contrast, the two-stage analysis is more practical for large-scale data but with some risk of missing possible pure epistasis [50]. More practical usage of both two-locus analysis modes in real case-control studies is required before the feasibility issue can be fully addressed.

Many genetic association studies reveal that various complex diseases are results of putative multi-locus interactions [11,46,53]. With the constraints on a computational capability, exhaustive multi-locus analysis in large-scale or genome-wide association studies would be infeasible [52]. On the other hand, single-locus analysis would be unsuitable for the detection of pure epistasis. One possible approach that provides a trade-off between a computational limitation and an epistasis detection capability is to capture a multi-locus interaction by combining multiple results from two-locus analysis. To achieve this, it is necessary to prove that once a multi-locus interaction model is broken down into a combination of two-locus models, all or some of these models remain detectable through two-locus analysis. Although it is hinted in an early work on two-locus analysis [52] that the proposed approach is plausible, explicit experimentation and testing has never been conducted.

In this article, the feasibility of employing an ensemble of two-locus analyses for the multi-locus interaction determination is demonstrated. Specifically, the significance of the two-locus analysis ensemble is assessed by an omnibus permutation test [54]. The proposed method is inspired by a set association approach [11], in which a limited number of sets that contain different numbers of SNPs are explored for possible association. These SNP sets are crucial in the global *p*-value calculation of the selected set via a permutation test and thus the decision to accept or reject the null hypothesis of no association. In other words, SNP set exploration and selection is required to assess the significance of the identified association. This means that the set association approach is equally interested in both SNP set selection and testing for significant association. The primary function of the proposed method is to detect possible association and assess its significance through the exploration of different ensembles of two-locus analyses. Hence, the proposed method is also equally interested in both ensemble selection and testing for significant association.

The proposed method is benchmarked against a simple exhaustive two-locus analysis technique, the set association approach [11], the correlation-based feature selection technique [14] and the tuned ReliefF technique [16]. These filter-based attribute selection techniques are suitable for the benchmark trial since they are capable of detecting association. The case-control classification models constructed from screened SNPs via a multifactor dimensionality reduction method [19] are also provided.

## Results and discussion

### Algorithm

**Figure 1:**
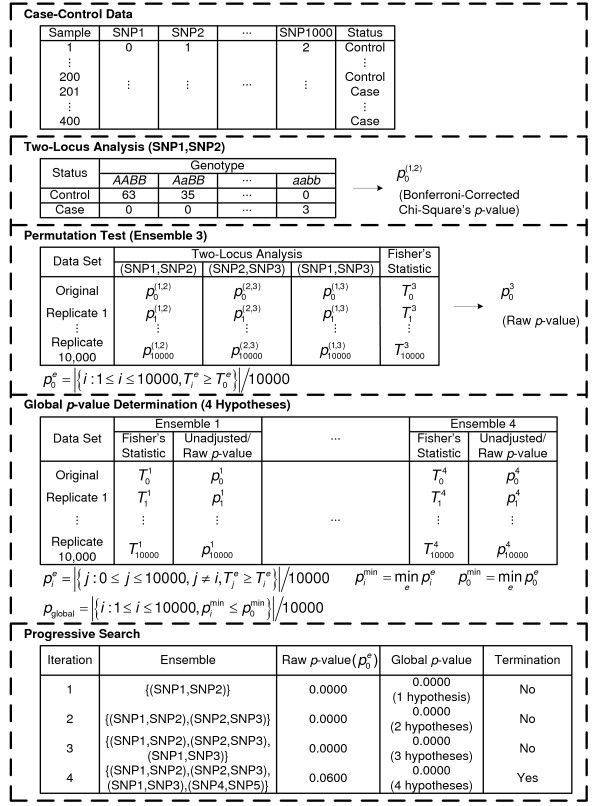
**Outline of 2LOmb**. In this example, the algorithm takes a balanced case-control data set that consists of 400 samples and 1,000 SNPs. Each genotype is represented by an integer: 0 denotes a homozygous wild-type genotype, 1 denotes a heterozygous genotype and 2 denotes a homozygous variant or homozygous mutant genotype. A *χ*^2 ^contingency table is then constructed for each pair of SNPs in two-locus analysis. This results in the total of  = 499,500 two-locus analyses. Thus, the Bonferroni-corrected *χ*^2^'s *p*-value for each two-locus analysis is the lower value between 499,500 × its uncorrected *p*-value and one. In one ensemble, Bonferroni-corrected *χ*^2^'s *p*-values from multiple two-locus analyses are combined together via a Fisher's combining function, which in turn provides a Fisher's test statistic result. The raw *p*-value for the ensemble is obtained through a permutation test, which is composed of 10,000 randomised permutation replicates. Since multiple ensembles may be tried during the identification of the best association explanation, a global *p*-value is calculated to account for multiple hypothesis testing. The global *p*-value is estimated through the same permutation test that gives the raw *p*-value for each ensemble. The progressive search for the best association explanation is carried out by incrementally adding a two-SNP unit to the current best ensemble. The condition for search termination is based on both the raw *p*-value for the explored ensemble and the global *p*-value. In this example, the search is terminated after the fourth ensemble is explored due to an increase in the raw *p*-value. Subsequently, the best SNP set for association explanation contains SNP1, SNP2 and SNP3 where the global *p*-value that accounts for testing of four hypotheses is *p *< 0.0001.

The proposed algorithm performs an omnibus permutation test on ensembles of two-locus analyses and is referred to as a 2LOmb technique. The algorithm consists of four steps as illustrated in Figure 1 and can be described as follows.

#### Two-locus analysis

Consider a case-control genetic association study with *n*_*m *_SNPs, for each pair of SNPs, a 2 × 9 contingency table with rows for disease status and columns for genotype configurations is created. A *χ*^2 ^test statistic and the corresponding *p*-value can subsequently be computed. With the total of *n*_*m *_SNPs, there are  = *n*_*m*_!/((*n*_*m *_- 2)!2!) possible SNP pairs. As a result, the *p*-value from each two-locus analysis must be adjusted by a Bonferroni correction. The Bonferroni-corrected *p*-value from each analysis is the lower value between  × the uncorrected *p*-value and one.

#### Permutation test

The *p*-value  for the null hypothesis  that ensemble *e*--an ensemble of two-locus analyses of interest--is not associated with the disease can be evaluated by a permutation test. To achieve this, a scalar statistic is first computed from a function that combines the Bonferroni-corrected *χ*^2^'s *p*-values of individual two-locus tests. A suitable combining function must (a) be non-increasing in each *p*-value, (b) attain its maximum value when any *p*-value equals to zero and (c) have a finite critical value that is less than its maximum for any significant level greater than zero [54]. In this study, a Fisher's combining function (-2∑_*i *_log(*p*_*i*_)) is selected [55]. The *p*-value for the ensemble of two-locus analyses is assessed via a permutation simulation. In each permutation replicate, samples are constructed such that the case/control status of each sample is randomly permuted while the total numbers of case and control samples remain unchanged. A *χ*^2 ^contingency table with new entries and a Bonferroni-corrected *p*-value for the two-locus analysis within each permutation replicate are then obtained. This, in turn, leads to a new Fisher's test statistic. Let  denote the value of Fisher's test statistic obtained for the *i*th permutation replicate,  is the fraction of permutation replicates with a test statistic greater than or equal to the test statistic obtained from the original case-control data (). In other words,

(1)

where *t *is the number of permutation replicates which is set to 10,000 in this study and |·| denotes the size of a set.

#### Global *p*-value determination

There are many candidate ensembles of two-locus analyses that can be explored. Let  be the global null hypothesis that none of *E *explored ensembles of two-locus analyses is associated with the disease, the test of the global null hypothesis leads to the global *p*-value and provides the genetic association explanation. In step 2, the *p*-value  for a fixed hypothesis  is a raw or unadjusted *p*-value. To account for the correlation among multiple hypotheses that have been tested during the exploration through many candidate ensembles, the testing result of the global null hypothesis depends on . In other words, the global null hypothesis is rejected if the minimum of the raw *p*-values is sufficiently small. The distribution of  can again be determined by a permutation simulation. However, a nested simulation is unnecessary since the same set of permutation replicates for the  determination can be reused in the estimation of the empirical distribution of [56]. This strategy has been successfully implemented in a number of genetic association detection techniques, including a set association approach [11] and a haplotype interaction approach embedded in FAMHAP [57,58]. The unadjusted *p*-value for the permutation replicate *i *of each hypothesis *e *is thus given by

(2)

Let  be the minimum of unadjusted *p*-values over all explored ensembles of two-locus analyses in the *i*th permutation replicate, the *p*-value for the global null hypothesis *H*_0 _is defined by

(3)

#### Search for the best ensemble of two-locus analyses

A simple progressive search is used to identify the best ensemble of two-locus analyses. The search begins by locating the best two-SNP unit with the smallest Bonferroni-corrected *χ*^2^'s *p*-value from step 1. A permutation test is then performed for this two-locus analysis, yielding both raw and global *p*-values since only one hypothesis has been explored. Next, the search attempts to combine the existing best two-SNP unit with the two-SNP unit that possesses the next smallest Bonferroni-corrected *χ*^2^'s *p*-value from step 1 and does not have a higher permutation *p*-value than the first two-SNP unit. If this new ensemble yields either a higher raw *p*-value or the same raw *p*-value but a higher global *p*-value from a permutation test, the search is terminated and the association is explained by the previously identified two-locus analysis. Otherwise, the best ensemble of two-locus analyses is updated and the process of appending more two-SNP units to the ensemble continues. The progressive search terminates when deterioration in the raw or global *p*-value is detected, or all possible two-locus analyses have been included in the ensemble. It is recalled from step 3 that for the best ensemble containing *E *- 1 < two-locus analyses, its global *p*-value is obtained from the evaluation of *E *hypotheses.

### Validity of the algorithm

A permutation replicate in 2LOmb is constructed by randomly assigning the case or control status to each sample while maintaining the original proportion of case and control samples. Once the construction of a permutation replicate is finished, the assigned case and control labels remain fixed to the samples. The pattern of case and control labels in each permutation replicate is thus constant and unique. Therefore, the Bonferroni-corrected *χ*^2^'s *p*-values from any two-SNP units within a permutation replicate are calculated from the same case-control data set. Hence, the combining of these Bonferroni-corrected *χ*^2^'s *p*-values via a Fisher's combining function is attainable. The calculation of Fisher's test statistics from all permutation replicates and the original data set leads to the raw or unadjusted *p*-value  for the null hypothesis  of the ensemble *e *as given in equation 1. Since the same set of permutation replicates is always used during the evaluation of each ensemble, the raw *p*-values for the null hypotheses from all ensembles can be directly compared against one another. Furthermore, the global *p*-value calculation is based on this set of permutation replicates. This is possible because the unadjusted *p*-value for the permutation replicate *i *of ensemble *e *or  can be calculated in a similar manner to the raw *p*-value  as defined in equation 2. The unadjusted *p*-values for the same permutation replicate but different ensembles can also be directly compared and the subsequent calculation of  is attainable. With  and , the *p*-value for the global null hypothesis  that incorporates all *E *explored hypotheses can be determined by equation 3. In summary, only one set of permutation replicates is required for the calculation of both the raw *p*-value for the null hypothesis of every ensemble and the global *p*-value. The *p*-values can be compared in each step of 2LOmb. Consequently, the selection of the best ensemble for association explanation can be carried out via a *p*-value comparison.

### Testing with simulated data

2LOmb is benchmarked against a simple exhaustive two-locus analysis technique, a set association approach (SAA) [11], a correlation-based feature selection (CFS) technique [14] and a tuned ReliefF (TuRF) technique [16] in a simulation trial. The exhaustive two-locus analysis is simply the two-locus analysis procedure from the first step of the 2LOmb algorithm. An interaction is declared if at least one two-SNP unit with a Bonferroni-corrected *χ*^2^'s *p*-value below 0.05 is detected. The exhaustive two-locus analysis reports all SNPs that meet this detection condition. The simulation covers two main data categories: null data of no significant genetic association and data with causative SNPs which signify pure epistasis. The algorithm performance on the null data provides an indication for the false-positive error. On the other hand, the algorithm performance on the data with causative SNPs indicates the detection capability. An efficient algorithm should produce an output with a low number of SNPs and a high number of correctly-identified causative SNPs when epistasis is present. Similarly, it should also report that there are no causative SNPs in the null data. These two measures on the number of SNPs in the results are used as the performance indicators.

Each simulated data set contains 1,000, 2,000 or 4,000 SNPs in which either there are no causative SNPs or there is pure epistasis, governed by two, three or four causative SNPs. The allele frequencies of all causative SNPs are 0.5 while the minor allele frequencies of the remaining SNPs are between 0.05 and 0.5. The data set consists of balanced case-control samples of sizes 400, 800 or 1,600. All SNPs in control samples are in Hardy-Weinberg equilibrium (HWE) [59]. The genotype distribution of causative interacting SNPs follows the pure epistasis model by Culverhouse et al. [42], leading to three interesting values of heritability: 0.01, 0.025 and 0.05. Every SNP in each data set exhibits no marginal single-locus effect (Bonferroni-corrected *χ*^2^'s *p*-value > 0.05). Twenty-five independent data sets for each simulation setting are generated via a genomeSIM package [60]. A paired *t*-test is suitable to assess the significance of results since the same simulated data sets are used during the algorithm benchmarking.

**Figure 2:**
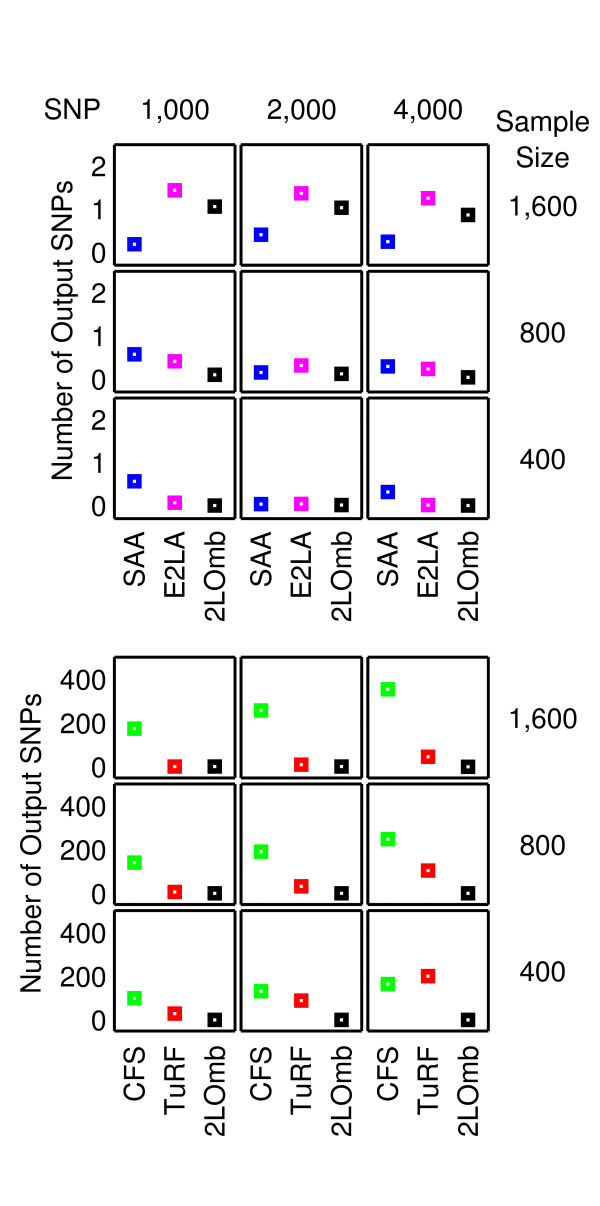
**Performance of the exhaustive two-locus analysis, SAA, CFS, TuRF and 2LOmb in the null data problem**. The results are averaged over 25 independent simulations. False detection is declared for the exhaustive two-locus analysis, SAA and 2LOmb if the *p*-values used as detection indicators in their results are less than 0.05. The results from the exhaustive two-locus analysis (E2LA), SAA, CFS, TuRF and 2LOmb are displayed using magenta, blue, green, red and black markers, respectively. In each chart, the horizontal axis represents the detection algorithm while the vertical axis represents the number of output SNPs reported by the algorithm. The top nine charts are displayed using a finer scale than the bottom nine charts.

**Figure 3:**
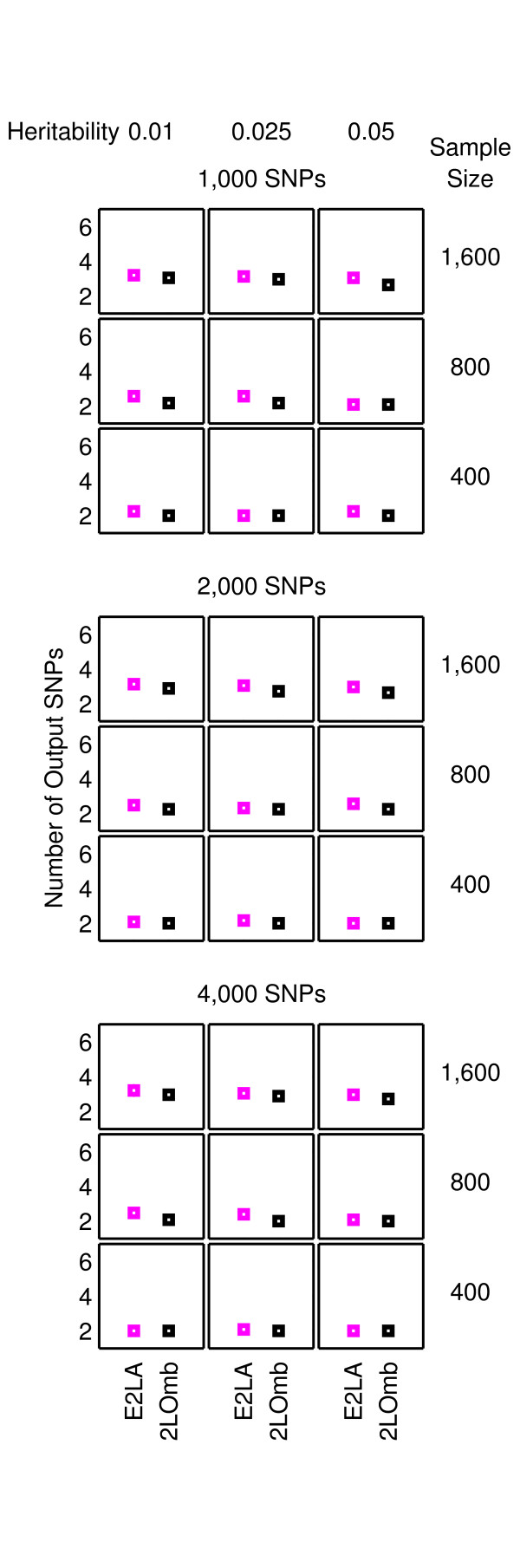
**Performance of the exhaustive two-locus analysis and 2LOmb in the two-locus interaction problem**. The results are averaged over 25 independent simulations. Detection is declared for the exhaustive two-locus analysis and 2LOmb if the *p*-values used as detection indicators in their results are less than 0.05. The results from the exhaustive two-locus analysis (E2LA) and 2LOmb are displayed using magenta and black markers, respectively. In each chart, the horizontal axis represents the detection algorithm while the vertical axis represents the number of output SNPs reported by the algorithm. All causative SNPs are present in outputs from both the exhaustive two-locus analysis and 2LOmb in all simulations.

**Figure 4:**
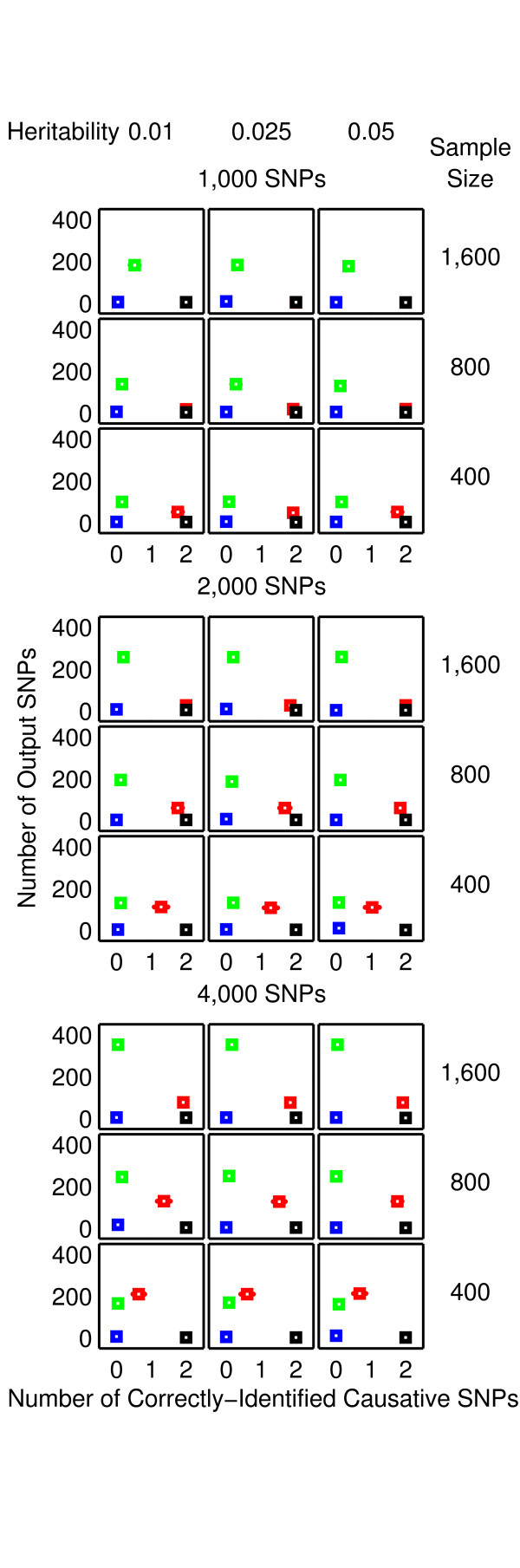
**Performance of SAA, CFS, TuRF and 2LOmb in the two-locus interaction problem**. The results are averaged over 25 independent simulations. Detection is declared for SAA and 2LOmb if the *p*-values used as detection indicators in their results are less than 0.05. The results from SAA, CFS, TuRF and 2LOmb are displayed using blue, green, red and black markers, respectively. In each chart, the horizontal axis represents the number of correctly-identified causative SNPs while the vertical axis represents the number of output SNPs reported by the algorithm. The charts on which the red markers are invisible denote the situations in which the performance of TuRF and 2LOmb is similar. The charts in this figure are displayed using a coarser scale than the charts in Figure 3.

**Figure 5:**
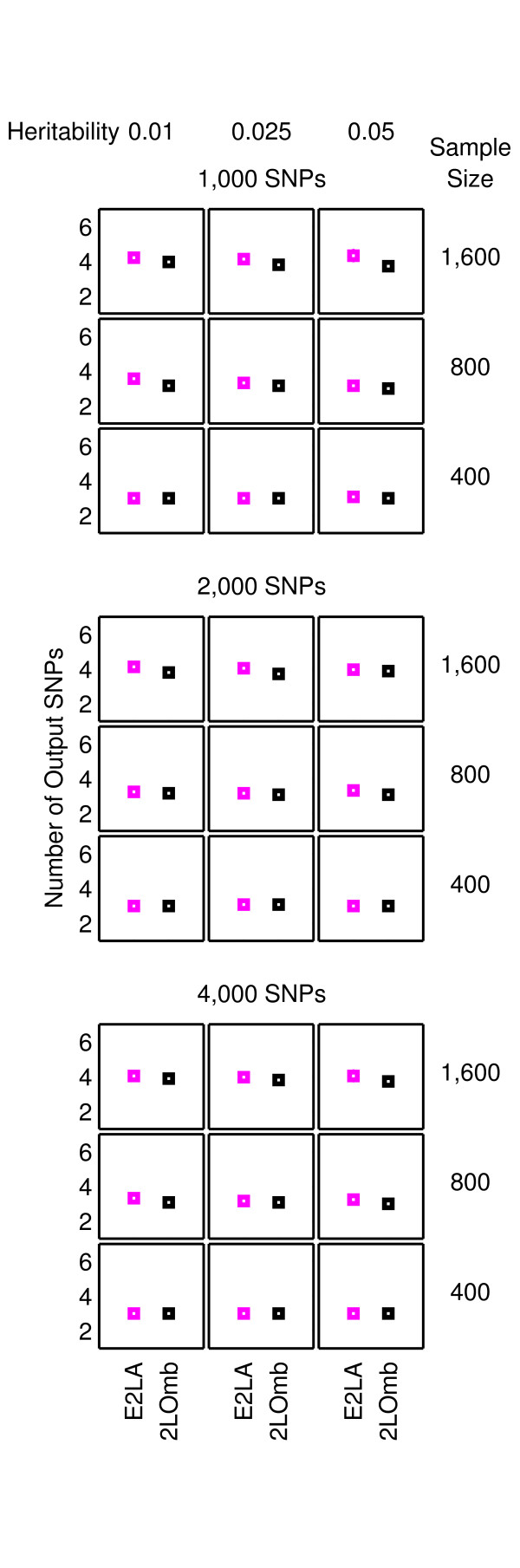
**Performance of the exhaustive two-locus analysis and 2LOmb in the three-locus interaction problem**. The explanation for how the results are obtained and displayed is the same as that given in Figure 3.

**Figure 6:**
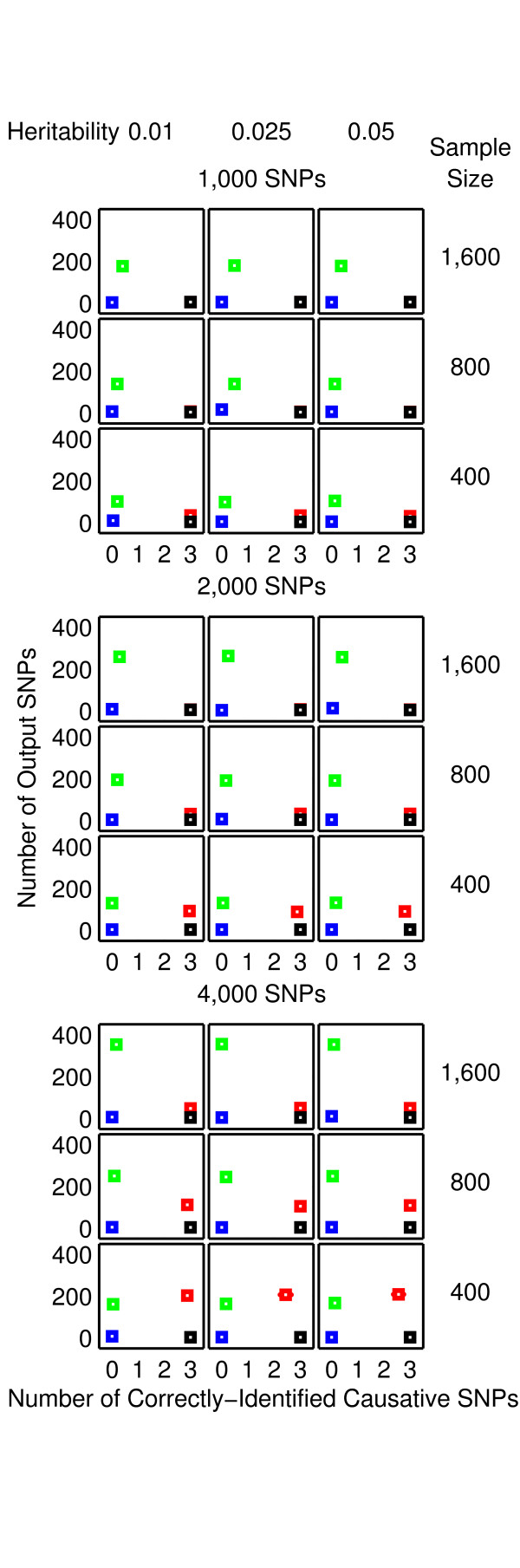
**Performance of SAA, CFS, TuRF and 2LOmb in the three-locus interaction problem**. The explanation for how the results are obtained and displayed is the same as that given in Figure 4.

**Figure 7:**
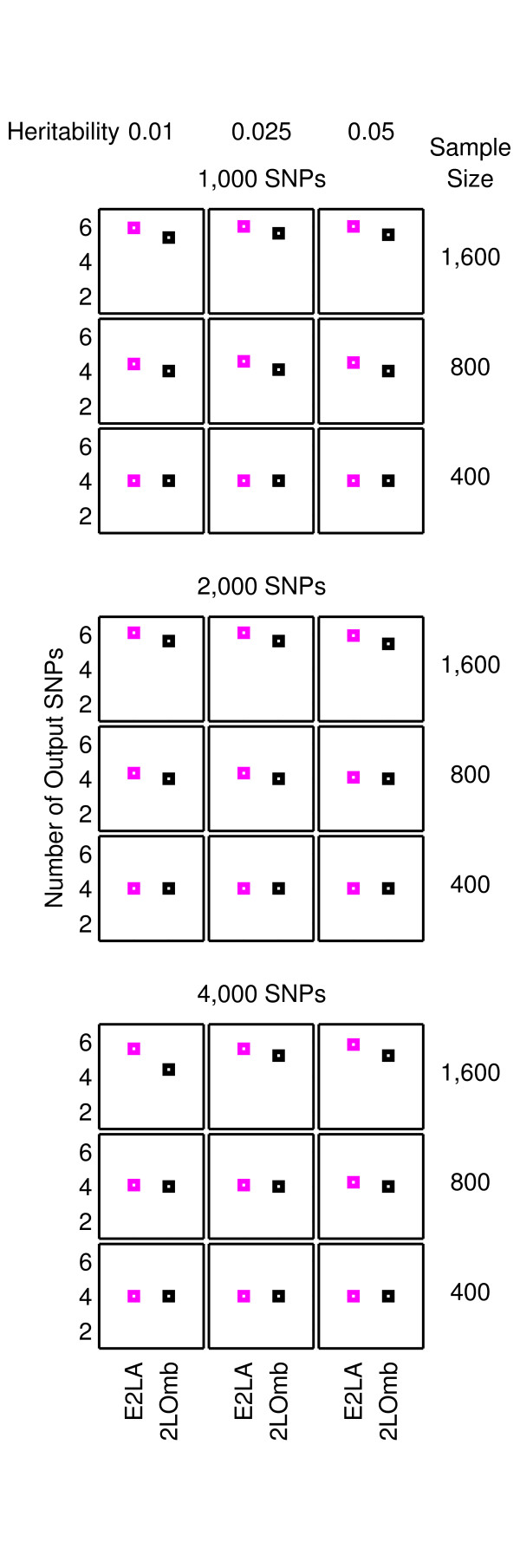
**Performance of the exhaustive two-locus analysis and 2LOmb in the four-locus interaction problem**. The explanation for how the results are obtained and displayed is the same as that given in Figure 3.

**Figure 8:**
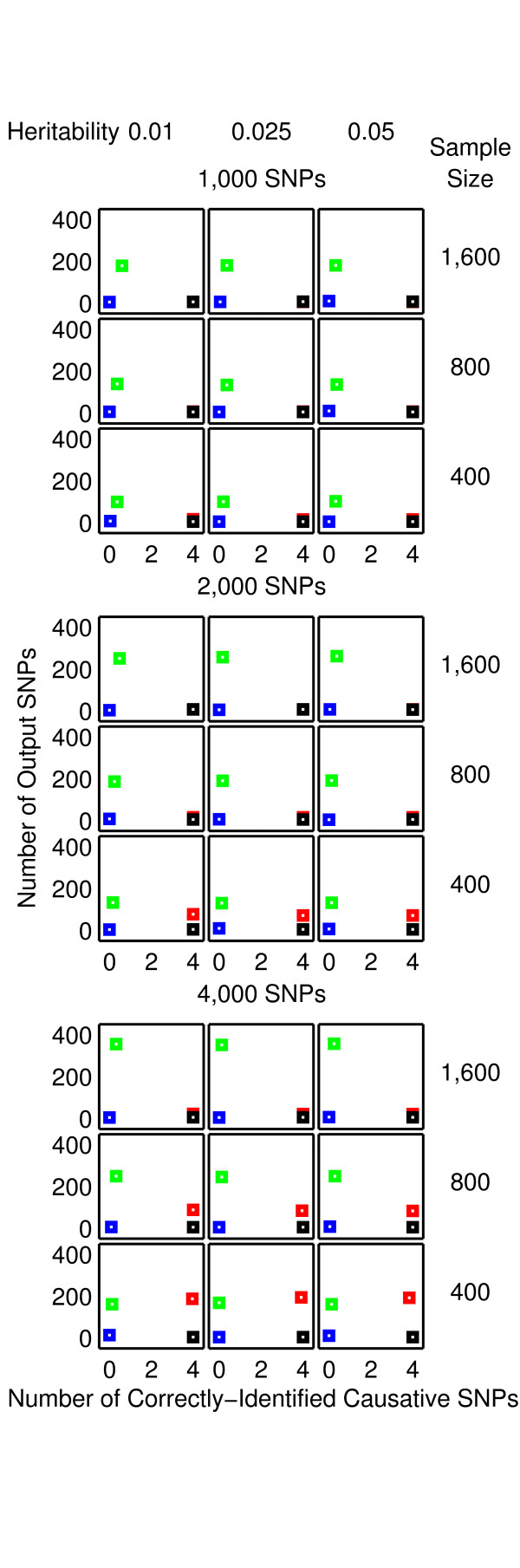
**Performance of SAA, CFS, TuRF and 2LOmb in the four-locus interaction problem**. The explanation for how the results are obtained and displayed is the same as that given in Figure 4.

The results from the null data problem are summarised in Figure 2 while the results from the two-, three-and four-locus interaction problems are shown in Figures 3-4, 5-6 and 7-8, respectively. Clearly, 2LOmb significantly outperforms other techniques in terms of the low number of output SNPs, the high number of correctly-identified causative SNPs or both in every interaction problem (a paired *t*-test on 675 benchmark results yields a *p*-value < 0.05). On the other hand, both 2LOmb and SAA have the lowest false-positive error when compared to other techniques in the null data problem (a paired *t*-test on 225 benchmark results yields a *p*-value < 0.05). The statistical power analysis also reveals that the benchmark trial with 25 independent data sets for each simulation setting is sufficient for an accurate evaluation of the overall algorithm performance (power > 0.95 for a Type I error rate of 0.05). These results can be further interpreted as follows.

The performance of many existing attribute selection techniques for pattern recognition depends on the level of attribute interactions. A number of techniques, including CFS, appear to function well under a moderate level of interactions. However, the performance of CFS appears to be significantly reduced when the interaction level becomes too high [14,61] because CFS favours an attribute that is strongly correlated with the classification outcome--disease status in this study--while at the same time is not correlated with other attributes. Since the main driving force behind epistasis is the interaction between SNPs, which are themselves attributes, CFS would not intuitively select all causative SNPs. Consequently, the SNP set produced by CFS appears to contain only uncorrelated SNPs. Obviously, a SNP that is a part of the interaction model would occasionally be picked up by CFS but CFS never successfully identifies all causative SNPs in any interaction problems. In addition, CFS reports more erroneous SNPs than other techniques in the null data problem and all three interaction problems due to many SNPs being uncorrelated.

The benchmarking of attribute selection techniques by Hall and Holmes [14] also reveals that ReliefF [15] is better than CFS in problems with a high level of interactions. Since ReliefF is essentially the core engine of TuRF, the results from this study are in agreement with the early benchmark trial. This finding strengthens the observation that the interaction level of SNPs in pure epistasis models is too high for CFS to handle. Similar to its predecessor, the performance of TuRF still depends on both the number of attributes and sample size. TuRF performs well in the majority of simulation scenarios with 1,000-2,000 SNPs and 800-1,600 samples. These scenarios are relatively easy since the number of SNPs is small while the sample size is large. However, the size of output SNP set, reported by TuRF from the null data problem and all three interaction problems, increases significantly when the difficulty level rises by either reducing the sample size or increasing the number of SNPs. This implies that when the problem contains a large number of candidate SNPs, the only way to ensure that TuRF reports a proper SNP set is to use a relatively large sample size, making it impractical in real genetic association studies due to many factors including disease prevalence, population size and genotyping cost.

The global *p*-values in most of the SAA results from the null data problem and all three interaction problems exceed 0.05, showing that SAA reports a low false-positive result in the null data problem. Nonetheless, SAA remains unsuitable for detecting pure epistasis because of its high false-negative error. This poor performance can be traced back to the manner in which SAA exploits an omnibus permutation test. As stated earlier, single-locus analysis does not detect any association between a SNP and the disease in this study. Hoh et al. [11] have demonstrated that genetic association can be more significantly observed when the single-locus test statistics are combined together. Nonetheless, there is an additional requirement that each causative SNP must exhibit a marginal single-locus effect. In the current study, the association signal from each causative SNP is lower than the required threshold, leading to similar test statistics and global *p*-values for both combinations of multiple SNPs which include causative SNPs and those which exclude causative SNPs.

Both 2LOmb and exhaustive two-locus analysis technique are capable of identifying all causative SNPs. However, the size of output SNP set from 2LOmb is significantly smaller than that from the exhaustive two-locus analysis. Appended SNPs to the causative SNPs in the output from 2LOmb and those from the exhaustive two-locus analysis are erroneous SNPs. These erroneous SNPs are parts of false two-SNP units with Bonferroni-corrected *χ*^2^'s *p*-values less than 0.05. A similar trend of results regarding the size of output SNP set is also observed in the benchmark trial involving the application of 2LOmb and exhaustive two-locus analysis to the null data. This signifies that the permutation test and the progressive search embedded in 2LOmb can help reducing the number of erroneous SNPs in the output.

As mentioned earlier, 2LOmb produces the best results among five techniques in the benchmark trial. 2LOmb has a low false-positive error in the null data problem and is capable of detecting all causative SNPs in every simulated data set in all three interaction problems. This performance is further strengthened by highly significant global *p*-values in 2LOmb results from all three interaction problems (*p *< 0.0001) and the presence of a SNP in common among some or all pairs of two-SNP units in the three- and four-locus interaction problems. Nonetheless, some of the 2LOmb outputs contain a few erroneous SNPs which are irrelevant to the correct association explanation. Since all three interaction problems involving different numbers of causative SNPs are investigated by varying the total number of SNPs, the sample size and the level of heritability, these parameters may influence the number of erroneous SNPs in the 2LOmb results. Similarly, the total number of SNPs and the sample size may affect the number of erroneous SNPs in the 2LOmb results from the null data problem. ANOVA reveals that the only source of variation that significantly affects the number of erroneous SNPs in the null data, two-locus interaction and three-locus interaction problems is the sample size (*p *< 0.000001). In addition, the sample size must be greater than 800 for an increase in the number of erroneous SNPs to be significant. In contrast, ANOVA reveals that two sources of variation that affect the number of erroneous SNPs in the four-locus interaction problem are the sample size (*p *< 0.000001) and the total number of SNPs (*p *< 0.00005). Similar to the null data, two-locus interaction and three-locus interaction problems, the sample size in the four-locus interaction problem must be greater than 800 to create a significant increase in the number of erroneous SNPs. On the other hand, the number of erroneous SNPs appears to decrease when the total number of SNPs increases. These two sources of variation also interact with each other (*p *< 0.005). However, the interaction is most evident only when the sample size is large, i.e. when the sample size is 1,600.

ANOVA shows that the number of erroneous SNPs in the 2LOmb results is influenced by the sample size and the total number of SNPs but not by the heritability. It is observed that the number of erroneous SNPs increases when the sample size is large. This counterintuitive phenomenon can be explained as follows. As 2LOmb combines *p*-values that are determined from *χ*^2 ^tests, the number of entries for the contingency table construction is large when the sample size is large. This subsequently leads to a significantly large *χ*^2 ^statistic and hence an extremely small *p*-value if the SNPs under consideration are causative SNPs. At the same time, the possibility that a reasonably large *χ*^2 ^statistic and a small *p*-value can be obtained by chance from a two-SNP unit which is irrelevant to the correct association explanation also inevitably increases. With the increase in the possibility of erroneous SNP inclusion, the size of output SNP set gets bigger when the sample size is large. Another observation that appears to be counterintuitive is the reduction in the number of erroneous SNPs when the total number of SNPs increases. This phenomenon is the result of the Bonferroni correction usage. When the total number of SNPs is doubled, the Bonferroni correction factor in 2LOmb is quadrupled. A higher correction factor leads to a more stringent criterion for SNP selection. This subsequently leads to the reduction in the number of erroneous SNPs when the total number of SNPs is large.

In contrast to the first two parameters, different levels of heritability appear to have no effect on the 2LOmb results because all simulated data sets have balanced case-control samples and the embedded interaction models have the same architecture. For instance, a two-locus interaction model leads to zero penetrances for genotypes *AABB*, *AABb*, *AaBB*, *Aabb*, *aaBb *and *aabb*. Hence, the penetrances for these six genotypes are always equal to zero regardless of the heritability. On the other hand, genotypes *AAbb*, *AaBb *and *aaBB *have non-zero penetrances (see Methods for details). Therefore, different heritability levels certainly lead to different penetrances for genotypes *AAbb*, *AaBb *and *aaBB*. However, the ratios between the penetrances of these three genotypes are fixed and independent of the heritability. This model description can be generalised to cover the other multi-locus interaction models. In addition, the maximum penetrance in any two-locus or multi-locus interaction models always stays below 0.1 even though the heritability is at the highest level (see Methods for details). This means that case samples are always over-sampled from affected individuals to achieve a balanced case-control data set. Since all explored heritability levels lead to the same case over-sampling pattern, the simulated data sets of which the only primary difference being the heritability levels are indistinguishable from one another. This leads to the result similarities in interaction problems with the same number of SNPs in the data set, sample size and number of causative SNPs but different levels of heritability as shown in Figures 3, 4, 5, 6, 7 and 8. The result trend is also independent of the number of simulated data sets used in the benchmark trial.

In a permutation test, the ability to differentiate between two *p*-values is influenced by the number of permutation replicates. With *t *permutation replicates, the test declares an actual *p*-value that is less than 1/*t *to be zero. During the progressive search for the best ensemble, the inclusion of a new two-SNP unit is accepted if this inclusion does not worsen the current result. If the number of permutation replicates is too low, the search may include erroneous two-SNP units that are irrelevant to the correct association explanation. The analysis is confirmed as the number of output SNPs from 2LOmb is equal to the number of causative SNPs in most of simulation results. This phenomenon suggests that the number of permutation replicates employed in this study (*t *= 10,000) is high enough to screen off most of the erroneous two-SNP units. In other words, the inclusion of these erroneous two-SNP units leads to an increase in the *p*-value by at least 1/*t*. Nonetheless, the fact that 2LOmb results are not entirely free from erroneous SNPs suggests that there are erroneous two-SNP units with extremely small *p*-values. It is advisable to perform a genotype relative risk calculation for the elimination of erroneous SNPs. If the presence of an erroneous two-SNP unit is suspected, its result on two-locus genotype relative risk would not be as significant as that from the other two-SNP units in the ensemble. Alternatively, an additional means for further SNP screening by other techniques such as MDR is also recommended. The chance of erroneous SNP discovery would be further minimised by employing two consecutive attribute selection techniques. The same concept has been adopted for the implementation of MDR software, in which many additional filters including a *χ*^2 ^test, an odds ratio test, ReliefF and TuRF are available for SNP screening prior to the MDR analysis.

**Figure 9:**
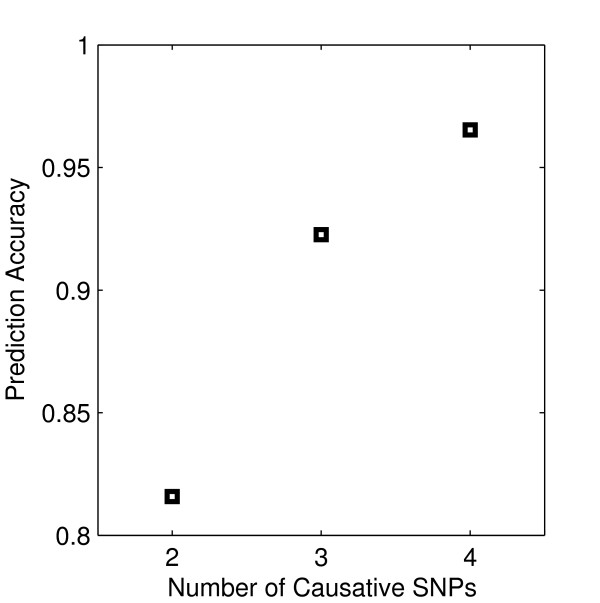
**Prediction accuracy from the MDR analysis**. A 10-fold cross-validation strategy is applied during the accuracy evaluation. The best MDR model is located by exploring all possible SNP combinations. All erroneous SNPs, which are left over after the screening by 2LOmb, have been successfully identified. All MDR models contain the correct number of causative SNPs. In addition, the MDR cross-validation consistency is 10/10.

**Figure 10:**
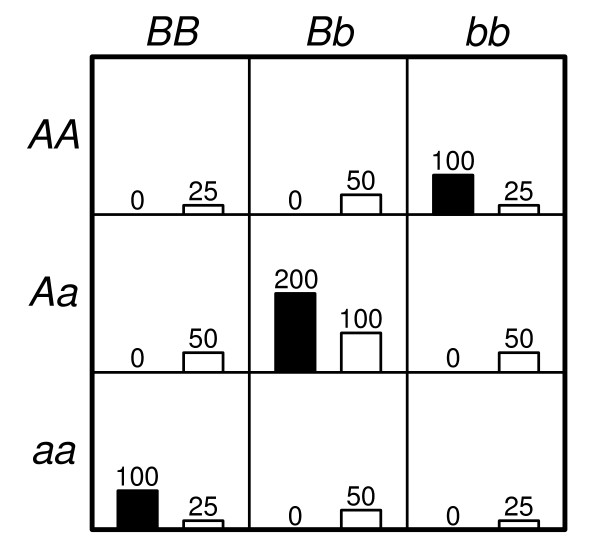
**Genotype distribution of two causative SNPs in a balanced case-control data set with the sample size of 800**. The left (black) bar in each cell represents the number of case samples while the right (white) bar represents the number of control samples. The cells with genotypes *AABB*, *AABb*, *AaBB*, *Aabb*, *aaBb *and *aabb *are labelled as protective genotypes while the cells with genotypes *AAbb*, *AaBb *and *aaBB *are labelled as disease-predisposing genotypes.

The two-, three- and four-locus interaction data sets which have been screened for causative SNPs by 2LOmb are subsequently subjected to MDR analysis. MDR has successfully identified all erroneous SNPs and the correct interaction models have been constructed from all data sets. The prediction accuracy from the MDR analysis is illustrated in Figure 9. It is noted that the prediction accuracy from all data sets is quite high due to the manner in which the pure epistasis model is defined [42]. Using the penetrance table for a two-locus interaction model with the heritability = 0.01 (see Methods for details), the two-locus genotype distribution of causative SNPs in a balanced case-control sample set from simulated data with 800 samples can be estimated and shown in Figure 10.

Six genotypes in Figure 10 namely *AABB*, *AABb*, *AaBB*, *Aabb*, *aaBb *and *aabb *are protective genotypes. In other words, a sample with one of these six genotypes is a control sample because the penetrances for these genotypes are zero. It is also noted that the control samples with all nine genotypes precisely follow the distribution as jointly described by independent single-locus genotype distribution from loci A and B. In contrast, three remaining genotypes in Figure 10 namely *AAbb*, *AaBb *and *aaBB *are labelled as disease-predisposing genotypes because the majority of samples with these three genotypes are case samples. Samples with these genotypes may be either case or control samples because the penetrances for these genotypes are between zero and one. In fact, the probabilities that persons with these genotypes to have the disease are quite low since the penetrances for these genotypes are small. However, case samples must be over-sampled from affected individuals to ensure a balanced case-control data set because the disease prevalence for this two-locus interaction model is only 0.004975. In addition, each case sample must contain one of these three genotypes because the penetrances for the other genotypes are zero. As a result, the case samples with these genotypes do not follow the same two-locus genotype distribution as in the control samples. With six genotypes being exclusively specific to control samples and the majority of three remaining genotypes being found in case samples, the MDR prediction accuracy for the two-locus interaction model is high. This explanation can also be generalised to cover the MDR results from the other multi-locus interaction data sets.

Another advantage of using 2LOmb for SNP screening prior to the MDR analysis is the reduction in computational time for interaction detection. The computational time for 2LOmb to finish screening the SNPs is provided to demonstrate this strength of 2LOmb. Moreover, the computational time required to identify causative SNPs by the MDR analysis and that by the combined approach which involves SNP screening by 2LOmb and follows by the MDR analysis is given. The previously-described simulated data sets with causative SNPs are used to produce the computational time results from the SNP screening by 2LOmb and the combined approach. All possible interaction models that can be constructed from the 2LOmb outputs are explored by MDR in the combined approach. On the other hand, the data sets for the direct MDR analysis are prepared by restricting the number of SNPs in each data set to 100. Only SNPs that are irrelevant to the correct association explanation are removed from the original simulated data sets. Furthermore, MDR only explores the interaction models that do not cover more than four SNPs in the data for this latter simulation setting. The summary of computational time required for the SNP screening by 2LOmb and that for both direct MDR and combined approaches to correctly identify all causative SNPs is given in Table 1. The maximum time required by 2LOmb to screen SNPs in the largest data set is 419 seconds or approximately seven minutes. Moreover, the combined 2LOmb and MDR approach discovers the correct causative SNPs much faster than MDR. This time reduction is achieved even though the problems have been simplified for the direct MDR analysis. A direct application of MDR to the original simulated data sets is certainly impractical.

**Table 1:** Computational time required by 2LOmb, a combined 2LOmb and MDR approach, and direct MDR analysis to detect interactions in simulated data sets with different sizes and different numbers of causative SNPs.

		**Computational Time Required by Each Approach (sec)**						
		**2LOmb**			**2LOmb+MDR**			**MDR**
**Number of Causative SNPs**	**Sample Size**	**1,000 SNPs**	**2,000 SNPs**	**4,000 SNPs**	**1,000 SNPs**	**2,000 SNPs**	**4,000 SNPs**	**100 SNPs**
2	400	15	37	135	17	39	137	7,656
	800	21	59	224	23	61	226	15,990
	1,600	36	106	400	38	108	402	31,222
3	400	22	43	140	24	45	142	7,721
	800	30	65	229	32	67	231	16,206
	1,600	50	115	406	52	117	408	31,232
4	400	32	55	150	34	57	152	7,841
	800	46	80	236	48	82	238	16,285
	1,600	70	133	419	72	135	421	31,637

Only one computing processor in a Beowulf cluster is occupied during the analysis of one data set. The test problems for the direct MDR analysis have been simplified by reducing the number of SNPs in each data set to achieve attainable computational time. The displayed time is collected from the processing of multiple independent data sets for each simulation setting. The computational time from the benchmark trial involving 2LOmb, and the combined 2LOmb and MDR approach is the maximum time needed by each method to detect interactions in one data set. In contrast, the computational time from the direct MDR analysis is the minimum time for the completion of interaction detection in one data set. The computational time required by 2LOmb for the null data problem is similar to that for the two-locus interaction problem.

The simulated multi-locus interaction problems in this article are based on the pure epistasis model by Culverhouse et al. [42]. It is possible to capture a number of multi-locus interactions with marginal two-locus effects via a combination of two-locus analyses. However, there are many multi-locus interaction scenarios without marginal two-locus effects. In such cases, 2LOmb and the exhaustive two-locus analysis technique are unable to detect interactions. Among the explored techniques, TuRF and MDR have a better chance of detection. Nonetheless, TuRF functions well only when the total number of SNPs in data is small and the sample size is large enough while the total number of SNPs in data affects the practicality of direct MDR analysis.

Every attribute selection technique has a limitation in terms of the maximum numbers of samples and attributes that it can handle. Single-locus analysis techniques always have a higher limit than multi-locus analysis techniques. Because attribute subset evaluation is usually integrated into multi-locus analysis techniques, consequently the number of possible attribute subsets that can be explored is extremely large when the candidate attribute set is large. Together with a potentially large sample size, a higher computational requirement for multi-locus analysis techniques is inevitable. As a result, the direct application of multi-locus analysis techniques to a much larger data set than those presented in this article, which is usually considered in genome-wide association studies, would be impractical. However, it is reasonable to expect that both marginal single-locus and epistatic effects are present in any genome-wide data sets. A multi-stage strategy that incorporates multiple techniques, designed for different detection modes, would be more suitable to handle large data. For instance, the marginal single-locus effects should be the first priority and, as such, be detected by single-locus analysis. Then, a special case of pure epistasis [2] or semi-purely epistatic events, in which a SNP displaying a marginal single-locus effect interacts with a SNP that exhibits no marginal single-locus effect, should be considered. Many two-locus analysis techniques have been proven to be well suited to this type of epistasis [40,50,51]. Finally, the detection of pure epistasis is carried out in the last stage. With the reduction of SNPs from the first stage, the chance that some multi-locus analysis techniques are applicable to the remaining SNPs increases. In addition to the multi-stage approach, a prior knowledge regarding the previously reported association can be exploited to select candidate genes based upon ontology and pathways. This practice is due to the necessity for the derivation of plausible interpretation. The screening for SNPs within or near candidate genes before the association detection also increases the chance that multi-locus analysis techniques can be applied to the remaining data.

### Testing with real data

2LOmb has been applied to study a type 2 diabetes mellitus (T2D) data set, collected and investigated by the Wellcome Trust Case Control Consortium (WTCCC) [3]. The data set consists of 1,999 case samples from affected individuals in the UK and 3,004 control samples, which are the results of a merging between 1,500 samples from the UK blood services and 1,504 samples from the 1958 British birth cohort. The original genome-wide data set contains 500,568 SNPs that are obtained through the Affymetrix GeneChip 500 K Mapping Array Set. The SNP set is primarily reduced by screening for SNPs within and near 372 candidate genes collected by the Human Genome Epidemiology Network (HuGENet) [62]. These candidate genes cover genes from both positive and negative genetic association reports, in which studies are conducted in various ethnic groups and populations. The SNP set is further reduced by removing SNPs that exhibit strong evidence of genetic association via single-locus analysis. The final SNP set contains 7,065 SNPs from 370 candidate genes. All SNPs in the reduced data set exhibit no marginal single-locus effects (Bonferroni-corrected *χ*^2^'s *p*-value > 0.05). Detailed description of the final SNP set is given in the supplement (see Additional file 1).

**Figure 11:**
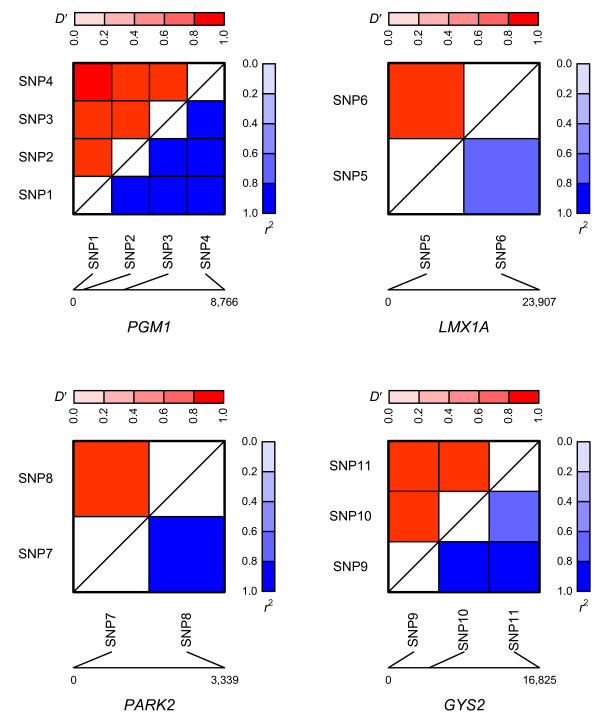
**Linkage disequilibrium (LD) patterns of SNPs in *PGM1*, *LMX1A*, *PARK2 *and *GYS2***. LD is explained via *D' *displayed in the upper triangle and *r*^2 ^displayed in the lower triangle. Dark colours indicate high values while pale colours indicate low values. Distances between SNPs are given in terms of the number of base pairs. SNP1 = rs2269241, SNP2 = rs2269239, SNP3 = rs3790857, SNP4 = rs2269238, SNP5 = rs2348250, SNP6 = rs6702087, SNP7 = rs1893551, SNP8 = rs6924502, SNP9 = rs6487236, SNP10 = rs1871142 and SNP11 = rs10770836.

**Figure 12:**
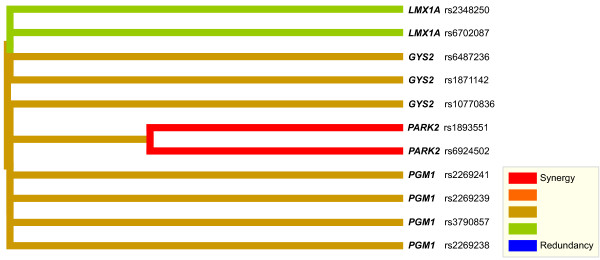
**Interaction dendrogram produced from 11 SNPs that are chosen by 2LOmb**. The colours in the dendrogram comprise a spectrum of colours representing a transition from synergy to redundancy. Synergy denotes the situation in which the entropy-based interaction between two SNPs provides more information than the entropy-based correlation between the pair. Redundancy refers to the situation in which the entropy-based interaction between two SNPs provides less information than the entropy-based correlation between the pair [7].

The 2LOmb search in the reduced T2D data set takes 3,456 seconds (57.6 minutes) of computational time on the Beowulf cluster. The possible genetic association is detected from 11 intronic SNPs in four genes (global *p*-value < 0.0001). Details of these SNPs, the two-SNP units that exhibit marginal two-locus effects and the identified genes are given in Table 2. A two-SNP unit is located in *LMX1A*. A two-SNP unit is also detected in *PARK2*. In addition, there is one SNP in common among SNPs in both *GYS2 *two-SNP units. Similarly, there is one common SNP among three two-SNP units located in *PGM1*. Nonetheless, a two-SNP unit in which each SNP is located in a different gene is absent, indicating that there is no evidence of gene-gene interactions which can be observed from the 2LOmb result. Linkage disequilibrium (LD) analysis is subsequently performed using a JLIN package [63] and the resulting LD patterns are illustrated in Figure 11. It is noted that there is strong LD among SNPs within each gene due to high values of *D' *[64] and *r*^2 ^[65]. The genotype and haplotype relative risks are then calculated and the results are presented in Tables 3, 4, 5, 6, 7, 8, 9 and 10. Haplotype inference is carried out using an expectation-maximisation method [66]. The analysis reveals that a more prominent indication of a relative risk is observed when two-SNP units are considered. It is also noted that the genotype relative risk is directly influenced by the haplotype relative risk once a genotype is phased into all possible haplotype pairs. The detection of these two-SNP units is thus believed to be the consequence of haplotype effects. An early T2D association study also reveals similar haplotype effects in FUSION data [67]. Next, an interaction dendrogram [68,69] constructed from the 11 SNPs by MDR software is given in Figure 12. A strong synergistic effect between the two SNPs in *PARK2 *is clearly observed. In contrast, the interactions between *PGM1*, *LMX1A*, *PARK2 *and *GYS2 *are clearly absent.

**Table 2:** 2LOmb identifies 11 intronic SNPs, which are located in four genes, from the reduced T2D data.

**Gene**	**Chromosome and Location**	**Two-SNP Unit in the Ensemble**
*PGM1 *(phosphoglucomutase 1)	1p31	(rs2269241, rs3790857)
		(rs2269239, rs3790857)
		(rs3790857, rs2269238)
*LMX1A *(LIM homeobox transcription factor 1, alpha)	1q22-q23	(rs2348250, rs6702087)
*PARK2 *(Parkinson disease (autosomal recessive, juvenile) 2, parkin)	6q25.2-q27	(rs1893551, rs6924502)
*GYS2 *(glycogen synthase 2 (liver))	12p12.2	(rs6487236, rs1871142)
		(rs1871142, rs10770836)

Association between these SNPs and the disease is possible (global *p*-value < 0.0001). Seven two-SNP units are present in the ensemble where each unit contains a pair of SNPs from the same gene.

**Table 3:** Genotype relative risk evaluated from genotype distribution of SNPs in *PGM1*.

		**Frequency**			
**SNP**	**Genotype**	**Case**	**Control**	**Relative Risk**	**95% CI**
SNP1	0	0.6513	0.6528	0.9977	(0.9573-1.0399)
	1	0.3082	0.3076	1.0018	(0.9204-1.0905)
	2	0.0405	0.0396	1.0229	(0.7757-1.3488)
SNP2	0	0.6493	0.6521	0.9957	(0.9552-1.0379)
	1	0.3087	0.3079	1.0024	(0.9209-1.0910)
	2	0.0420	0.0399	1.0519	(0.8006-1.3822)
SNP3	0	0.6153	0.6568	0.9368	(0.8972-0.9782)
	1	0.3472	0.3056	1.1361	(1.0479-1.2316)
	2	0.0375	0.0376	0.9974	(0.7490-1.3281)
SNP4	0	0.6638	0.6668	0.9956	(0.9564-1.0364)
	1	0.2991	0.2969	1.0074	(0.9237-1.0988)
	2	0.0370	0.0363	1.0202	(0.7636-1.3631)
(SNP1, SNP3)	0 0	0.6043	0.6448	0.9372	(0.8966-0.9796)
	**0 1**	**0.0470**	**0.0080**	**5.8858**	**(3.7730-9.1816)**
	1 0	0.0110	0.0120	0.9183	(0.5420-1.5561)
	1 1	0.2961	0.2943	1.0064	(0.9222-1.0983)
	1 2	0.0010	0.0013	0.7514	(0.1378-4.0984)
	2 1	0.0040	0.0033	1.2022	(0.4753-3.0408)
	2 2	0.0365	0.0363	1.0064	(0.7523-1.3463)
(SNP2, SNP3)	00	0.6038	0.6448	0.9364	(0.8958-0.9789)
	**01**	**0.0455**	**0.0073**	**6.2159**	**(3.9154-9.8681)**
	10	0.0115	0.0117	0.9875	(0.5853-1.6661)
	11	0.2966	0.2949	1.0058	(0.9218-1.0975)
	12	0.0005	0.0013	0.3757	(0.0420-3.3588)
	21	0.0050	0.0033	1.5028	(0.6266-3.6038)
	22	0.0370	0.0363	1.0202	(0.7636-1.3631)
(SNP3, SNP4)	00	0.6138	0.6551	0.9369	(0.8971-0.9785)
	01	0.0015	0.0017	0.9017	(0.2157-3.7686)
	**10**	**0.0500**	**0.0117**	**4.2936**	**(2.9340-6.2831)**
	11	0.2971	0.2936	1.0121	(0.9274-1.1044)
	21	0.0005	0.0017	0.3006	(0.0351-2.5706)
	22	0.0370	0.0360	1.0297	(0.7702-1.3765)

The relative risk is calculated from the ratio between the probability of a genotype of interest occurring in the case group and that of the same genotype occurring in the control group. Only the relative risks based on genotypic information from each SNP and SNP pairs identified by 2LOmb are considered. Characters 0, 1 and 2 represent different genotypes at each locus where 0 denotes a homozygous wild-type genotype, 1 denotes a heterozygous genotype and 2 denotes a homozygous variant or homozygous mutant genotype. The relative risk displayed in boldface is statistically significant. The major/minor alleles for rs2269241, rs2269239, rs3790857 and rs2269238 are T/C, G/C, C/T and G/T, respectively. The allelic information is extracted from the original T2D data. SNP1 = rs2269241, SNP2 = rs2269239, SNP3 = rs3790857 and SNP4 = rs2269238.

**Table 4:** Haplotype relative risk evaluated from genotype distribution of SNPs in *PGM1*.

		**Frequency**			
**SNP**	**Allele and Haplotype**	**Case**	**Control**	**Relative Risk**	**95% CI**
SNP1	0	0.8054	0.8066	0.9985	(0.9791-1.0183)
	1	0.1946	0.1934	1.0061	(0.9274-1.0916)
SNP2	0	0.8037	0.8061	0.9970	(0.9775-1.0168)
	1	0.1963	0.1939	1.0126	(0.9336-1.0982)
SNP3	0	0.7889	0.8096	0.9744	(0.9550-0.9943)
	1	0.2111	0.1904	1.1087	(1.0240-1.2003)
SNP4	0	0.8134	0.8152	0.9977	(0.9789-1.0170)
	1	0.1866	0.1848	1.0100	(0.9288-1.0982)
(SNP1, SNP3)	0 0	0.7812	0.8019	0.9742	(0.9543-0.9945)
	**0 1**	**0.0242**	**0.0047**	**5.1533**	**(3.3946-7.8231)**
	1 0	0.0077	0.0077	1.0000	(0.6349-1.5752)
	1 1	0.1869	0.1857	1.0064	(0.9257-1.0941)
(SNP2, SNP3)	00	0.7804	0.8017	0.9734	(0.9535-0.9938)
	**01**	**0.0232**	**0.0044**	**5.3216**	**(3.4557-8.1949)**
	10	0.0085	0.0079	1.0758	(0.6930-1.6701)
	11	0.1879	0.1861	1.0099	(0.9291-1.0977)
(SNP3, SNP4)	00	0.7881	0.8086	0.9747	(0.9552-0.9946)
	01	0.0008	0.0010	0.7661	(0.1943-3.0205)
	**10**	**0.0253**	**0.0067**	**3.7936**	**(2.6367-5.4582)**
	11	0.1858	0.1837	1.0113	(0.9298-1.0999)

The relative risk is calculated from the ratio between the probability of a haplotype of interest occurring in the case group and that of the same haplotype occurring in the control group. Haplotype inference is carried out using an expectation-maximisation method. Only the relative risks based on genotypic information from each SNP and SNP pairs identified by 2LOmb are considered. Characters 0 and 1 represent different alleles at each locus where 0 and 1 denote major and minor alleles, respectively. The relative risk displayed in boldface is statistically significant. The allelic information for each SNP is given in Table 3. SNP1 = rs2269241, SNP2 = rs2269239, SNP3 = rs3790857 and SNP4 = rs2269238.

**Table 5:** Genotype relative risk evaluated from genotype distribution of SNPs in *LMX1A*.

		**Frequency**			
**SNP**	**Genotype**	**Case**	**Control**	**Relative Risk**	**95% CI**
SNP5	0	0.8429	0.8642	0.9754	(0.9526-0.9987)
	1	0.1531	0.1315	1.1642	(1.0140-1.3366)
	2	0.0040	0.0043	0.9248	(0.3840-2.2271)
SNP6	0	0.8799	0.8492	1.0362	(1.0135-1.0594)
	1	0.1161	0.1465	0.7924	(0.6829-0.9193)
	2	0.0040	0.0043	0.9248	(0.3840-2.2271)
(SNP5, SNP6)	00	0.8329	0.8299	1.0036	(0.9784-1.0295)
	**01**	**0.0100**	**0.0343**	**0.2918**	**(0.1814-0.4695)**
	**10**	**0.0470**	**0.0193**	**2.4355**	**(1.7644-3.3618)**
	11	0.1061	0.1119	0.9482	(0.8061-1.1153)
	22	0.0040	0.0040	1.0018	(0.4103-2.4465)

The explanation for how the relative risks are obtained and displayed is the same as that given in Table 3. The major/minor alleles for rs2348250 and rs6702087 are G/A and G/C, respectively. The allelic information is extracted from the original T2D data. SNP5 = rs2348250 and SNP6 = rs6702087.

**Table 6:** Haplotype relative risk evaluated from genotype distribution of SNPs in *LMX1A*.

		**Frequency**			
**SNP**	**Allele and Haplotype**	**Case**	**Control**	**Relative Risk**	**95% CI**
SNP5	0	0.9195	0.9299	0.9887	(0.9774-1.0002)
	1	0.0805	0.0701	1.1494	(0.9997-1.3214)
SNP6	0	0.9380	0.9224	1.0168	(1.0059-1.0279)
	1	0.0620	0.0776	0.7997	(0.6892-0.9280)
(SNP5, SNP6)	00	0.9143	0.9124	1.0021	(0.9898-1.0145)
	**01**	**0.0051**	**0.0175**	**0.2931**	**(0.1829-0.4697)**
	**10**	**0.0236**	**0.0100**	**2.3640**	**(1.7150-3.2585)**
	11	0.0569	0.0601	0.9472	(0.8064-1.1127)

The explanation for how the relative risks are obtained and displayed is the same as that given in Table 4. The allelic information for each SNP is given in Table 5. SNP5 = rs2348250 and SNP6 = rs6702087.

**Table 7:** Genotype relative risk evaluated from genotype distribution of SNPs in *PARK2*.

		**Frequency**			
**SNP**	**Genotype**	**Case**	**Control**	**Relative Risk**	**95% CI**
SNP7	0	0.4802	0.5110	0.9398	(0.8873-0.9954)
	1	0.4322	0.4041	1.0695	(1.0008-1.1429)
	2	0.0875	0.0849	1.0313	(0.8581-1.2395)
SNP8	0	0.4892	0.4923	0.9937	(0.9380-1.0527)
	1	0.4087	0.4121	0.9917	(0.9267-1.0613)
	2	0.1021	0.0955	1.0682	(0.9009-1.2665)
(SNP7, SNP8)	00	0.4492	0.4844	0.9275	(0.8726-0.9858)
	01	0.0285	0.0260	1.0982	(0.7841-1.5380)
	02	0.0025	0.0007	3.7569	(0.7296-19.3450)
	**10**	**0.0400**	**0.0080**	**5.0092**	**(3.1856-7.8767)**
	11	0.3792	0.3848	0.9854	(0.9169-1.0590)
	12	0.0130	0.0113	1.1492	(0.6918-1.9089)
	21	0.0010	0.0013	0.7514	(0.1378-4.0984)
	22	0.0865	0.0836	1.0358	(0.8606-1.2465)

The explanation for how the relative risks are obtained and displayed is the same as that given in Table 3. The major/minor alleles for rs1893551 and rs6924502 are G/A and T/C, respectively. The allelic information is extracted from the original T2D data. SNP7 = rs1893551 and SNP8 = rs6924502.

**Table 8:** Haplotype relative risk evaluated from genotype distribution of SNPs in *PARK2*.

		**Frequency**			
**SNP**	**Allele and Haplotype**	**Case**	**Control**	**Relative Risk**	**95% CI**
SNP7	0	0.6963	0.7130	0.9766	(0.9515-1.0023)
	1	0.3037	0.2870	1.0582	(0.9950-1.1254)
SNP8	0	0.6936	0.6984	0.9931	(0.9672-1.0198)
	1	0.3064	0.3016	1.0159	(0.9563-1.0793)
(SNP7, SNP8)	00	0.6726	0.6937	0.9696	(0.9434-0.9966)
	01	0.0238	0.0194	1.2248	(0.9369-1.6011)
	**10**	**0.0210**	**0.0048**	**4.4215**	**(2.8972-6.7480)**
	11	0.2826	0.2822	1.0016	(0.9397-1.0675)

The explanation for how the relative risks are obtained and displayed is the same as that given in Table 4. The allelic information for each SNP is given in Table 7. SNP7 = rs1893551 and SNP8 = rs6924502.

**Table 9:** Genotype relative risk evaluated from genotype distribution of SNPs in *GYS2*.

		**Frequency**			
**SNP**	**Genotype**	**Case**	**Control**	**Relative Risk**	**95% CI**
SNP9	0	0.6473	0.6575	0.9846	(0.9447-1.0262)
	1	0.3167	0.3046	1.0396	(0.9558-1.1308)
	2	0.0360	0.0379	0.9491	(0.7105-1.2679)
SNP10	0	0.5933	0.6441	0.9211	(0.8805-0.9634)
	1	0.3712	0.3169	1.1713	(1.0839-1.2657)
	2	0.0355	0.0389	0.9119	(0.6828-1.2180)
SNP11	0	0.6058	0.6142	0.9864	(0.9427-1.0321)
	1	0.3507	0.3352	1.0461	(0.9675-1.1310)
	2	0.0435	0.0506	0.8601	(0.6650-1.1126)
(SNP9, SNP10)	00	0.5863	0.6335	0.9255	(0.8841-0.9689)
	**01**	**0.0610**	**0.0240**	**2.5463**	**(1.9135-3.3885)**
	10	0.0055	0.0107	0.5166	(0.2610-1.0224)
	11	0.3092	0.2919	1.0590	(0.9717-1.1540)
	12	0.0020	0.0020	1.0018	(0.2831-3.5456)
	21	0.0010	0.0010	1.0018	(0.1676-5.9902)
	22	0.0335	0.0370	0.9071	(0.6734-1.2218)
(SNP10, SNP11)	00	0.5463	0.5922	0.9224	(0.8776-0.9695)
	01	0.0455	0.0506	0.8997	(0.6982-1.1593)
	02	0.0015	0.0013	1.1271	(0.2525-5.0304)
	**10**	**0.0595**	**0.0220**	**2.7095**	**(2.0164-3.6408)**
	11	0.3032	0.2823	1.0739	(0.9839-1.1722)
	12	0.0085	0.0126	0.6723	(0.3805-1.1877)
	21	0.0020	0.0023	0.8587	(0.2517-2.9296)
	22	0.0335	0.0366	0.9153	(0.6791-1.2336)

The explanation for how the relative risks are obtained and displayed is the same as that given in Table 3. The major/minor alleles for rs6487236, rs1871142 and rs10770836 are A/G, G/A and G/A, respectively. The allelic information is extracted from the original T2D data. SNP9 = rs6487236, SNP10 = rs1871142 and SNP11 = rs10770836.

**Table 10:** Haplotype relative risk evaluated from genotype distribution of SNPs in *GYS2*.

		**Frequency**			
**SNP**	**Allele and Haplotype**	**Case**	**Control**	**Relative Risk**	**95% CI**
SNP9	0	0.8057	0.8098	0.9949	(0.9757-1.0146)
	1	0.1943	0.1902	1.0216	(0.9412-1.1087)
SNP10	0	0.7789	0.8026	0.9705	(0.9505-0.9908)
	1	0.2211	0.1974	1.1201	(1.0367-1.2102)
SNP11	0	0.7811	0.7818	0.9992	(0.9782-1.0205)
	1	0.2189	0.2182	1.0030	(0.9299-1.0818)
(SNP9, SNP10)	00	0.7740	0.7967	0.9715	(0.9512-0.9922)
	**01**	**0.0317**	**0.0131**	**2.4258**	**(1.8357-3.2056)**
	10	0.0049	0.0059	0.8330	(0.4807-1.4433)
	11	0.1894	0.1843	1.0276	(0.9455-1.1169)
(SNP10, SNP11)	00	0.7494	0.7692	0.9742	(0.9524-0.9965)
	01	0.0295	0.0334	0.8843	(0.7069-1.1061)
	**10**	**0.0318**	**0.0126**	**2.5275**	**(1.9064-3.3511)**
	11	0.1894	0.1848	1.0244	(0.9426-1.1134)

The explanation for how the relative risks are obtained and displayed is the same as that given in Table 4. The allelic information for each SNP is given in Table 9. SNP9 = rs6487236, SNP10 = rs1871142 and SNP11 = rs10770836.

Since many early genetic association studies of T2D and metabolic syndrome employ MDR analysis [43-45,47], additional MDR analysis would be useful for the comparison. The screened T2D case-control data set which contains 11 SNPs identified by 2LOmb is further subjected to MDR analysis. The prediction accuracy of the best MDR model is summarised in Table 11. The model covers six SNPs in three genes: *PGM1*, *PARK2 *and *GYS2*. These SNPs are also present in three two-SNP units identified by 2LOmb. It is noted that the prediction accuracy in this real data set is much less than that from the simulated data sets. Nevertheless, the attainment of low prediction accuracy does not necessarily suggest that there is no genetic association. Early works involving genetic association studies of T2D and metabolic syndrome in various populations via MDR analysis produce similar values of prediction accuracy as summarised in Table 12. The prediction accuracy by MDR from most studies is in the range of 0.5-0.6. The only genetic association study of T2D that the prediction accuracy is distinctively high is conducted in a Korean population [43]. Differences in genetic background, candidate genes and selected SNPs are the main causes of variation in the genetic association results. Although MDR does not select five SNPs from the 2LOmb output, these SNPs should not be regarded as erroneous SNPs because there is strong linkage disequilibrium among SNPs in each gene. Moreover, early genotype and haplotype relative risk analysis clearly indicates that each gene, identified by 2LOmb, plays a role in the T2D association explanation. Overall, the analysis with the methods above only confirms the positive association for *PGM1*, *LMX1A*, *PARK2 *and *GYS2 *while gene-gene interactions are clearly absent. This signifies that, for the current study, there is no interaction between each pair of the identified genes that can be described by purely epistatic two-locus interaction models. In addition, there are no interactions between these four genes that can be described by purely epistatic multi-locus interaction models with marginal two-locus effects.

**Table 11:** Prediction accuracy of the best MDR model constructed from the 2LOmb output.

**Description**	**Value**
SNP and Gene	rs2269241 (*PGM1*), rs3790857 (*PGM1*), rs1893551 (*PARK2*), rs6924502 (*PARK2*), rs1871142 (*GYS2*), rs10770836 (*GYS2*)
Classification (Training) Accuracy	0.5709
Prediction Accuracy	0.5402
Cross-Validation Consistency (CVC)	9/10

The model contains six SNPs from *PGM1*, *PARK2 *and *GYS2*. A permutation test with 1,000 randomised replicates of case-control data for this model reveals that the empirical *p*-value for the null hypothesis of no association is *p *< 0.001.

**Table 12:** Summary of prediction accuracy by MDR from early genetic association studies of T2D in a Korean population, a Han Chinese population from Taiwan, a female population from the US, and that from an early genetic association study of metabolic syndrome in an Italian population from the Centre East Coast Italy.

**Reference**	**Population**	**Gene**	**Prediction Accuracy**	**CVC**	**Permutation *p*-value**
Cho et al. [43]	Korean	*PPARG*, *UCP2*	0.7957	9/10	0.01
Hsieh et al. [44]	Han Chinese	*RXRG*, *EGFR*	0.6270	11/12	N/A
Qi et al. [45]	US	*KCNJ11*, *HNF4A*	0.5420	10/10	0.010
Fiorito et al. [47]	Italian	*PPARG*, *DIO2*	0.6170	10/10	0.005

A permutation test with 1,000 randomised replicates is performed to obtain the empirical *p*-value for the null hypothesis of no association in the studies conducted in the US and Italian populations. In contrast, a permutation test with 100 randomised replicates is performed to obtain the empirical *p*-value in the study conducted in the Korean population.

The four genes selected by 2LOmb regulate many pathways that involve in the disease development [70-72]. The genetic association studies involving these genes have been previously conducted in different populations. For instance, *LMX1A *has been chosen as a positional and biological candidate gene for a case-control study of T2D in Pima Indians [73]. This gene is chosen as a candidate because a linkage of T2D to chromosome 1q21-q23 has been previously reported [74]. In addition, *LMX1A *is one of LIM homeobox genes that are expressed in pancreas and has been shown to activate insulin gene transcription. Although SNPs have been carefully selected from the entire gene, no association between these SNPs in *LMX1A *and T2D has been found in this ethnic group.

*PARK2 *is another candidate gene that is also selected for case-control studies, based on evidence from genome-wide linkage analysis [75]. A linkage of T2D in an African American population to chromosome 6q24-q27 has been previously identified [76]. Although *PARK2 *mainly involves in the development of Parkinson's disease, single-locus analysis reveals strong evidence of association between SNPs, which are in the vicinity of SNPs identified by 2LOmb, and T2D in African Americans.

In contrast to *LMX1A *and *PARK2*, which are candidate genes in typical T2D case-control studies, *GYS2 *is considered in a study to identify genes responsible for troglitazone-associated hepatotoxicity in Japaneses with T2D [77]. In other words, both case and control samples in the study are drawn from troglitazone-treated T2D patients, in which case patients exhibit an abnormal increase in alanine transaminase (ALT) and aspartate transaminase (AST) levels. *GYS2 *regulates starch and sucrose metabolism and an insulin signalling pathway. The selected SNPs in *GYS2 *are not found to associate with troglitazone-induced hepatotoxicity.

Similar to the study of *GYS2*, the association study involving *PGM1 *is not carried out as a typical T2D case-control study. In fact, an attempt to identify association between *PGM1 *polymorphisms and obesity has been conducted among T2D affected individuals in Italy [78]. *PGM1 *regulates glycolysis and gluconeogenesis, starch and sucrose metabolism, galactose metabolism, a pentose phosphate pathway, and streptomycin biosynthesis. Isozyme polymorphisms [79,80], which are defined through structural differences in PGM1 protein, are used instead of SNPs in the study where positive association is identified.

In summary, positive association has been reported from previous studies involving *PARK2 *in African Americans and *PGM1 *in Italians. In contrast, negative association has been reported from previous studies about *LMX1A *in Pima Indians and *GYS2 *in Japaneses. Both *GYS2 *and *PGM1 *regulate starch and sucrose metabolism while *LMX1A *and *PARK2 *govern insulin gene transcription and Parkinson's disease development, respectively. The above discussion strengthens the importance of conducting large-scale association studies due to two main reasons. Firstly, a gene that does not contribute to the aetiology of a complex disease in one population may be important for association explanation in another population. Secondly, the absence of interacting candidate genes from a study may lead to negative association due to a lack of necessary genetic information. A two-locus interaction can occur between SNPs from genes that regulate one specific pathway [44] or between SNPs from genes that regulate different pathways [45]. Furthermore, a multi-locus interaction may involve both SNPs from genes that regulate the same pathway and SNPs from genes that govern different pathways. Hence, candidate genes should be selected by considering all pathways that directly and indirectly contribute to the disease development.

This study produces evidence of association between 11 intronic SNPs in *PGM1*, *LMX1A*, *PARK2 *and *GYS2*, and T2D in a UK population. Although there are other independent genome-wide T2D data sets, the association detection within these data using a similar methodology to the presented method has never been attempted because the methodology employed in the majority of genome-wide association studies is based on single-locus analysis [3,81]. It is recalled that each SNP explored in the reduced T2D data set exhibits no marginal single-locus effect. Hence, the most logical approach to confirm the possibility of replicating association results from the current study is to perform the same detection method on these independent data sets. This is certainly important to gain further understanding of the genetic role in T2D susceptibility.

### Implementation

2LOmb is implemented in a C programming language. All functions within the program are written by the first author except the *χ*^2 ^distribution function, which is taken from the Numerical Recipes in C [82]. The program can be compiled by Microsoft Visual Studio and GNU C compilers. The program has been successfully tested for the execution under Windows and Linux operating systems. The time required by 2LOmb to complete a problem containing *n *attributes is *T *(*n*) =  = *n*!*/*((*n *- 2)!2!) = *n*(*n *- 1)*/*2. 2LOmb thus has the order of *n*^2 ^time complexity (*T *(*n*) ∈ *O*(*n*^2^)). Consequently, 2LOmb can tackle problems in quadratic time. 2LOmb in its present form occupies one processor during the program execution. A parallel version of 2LOmb for genome-wide data is under development. All results included in the study are collected from the execution of computer programs in a Beowulf cluster. The computational platform consists of 12 nodes. Each node is equipped with dual Xeon 2.8 GHz processors and 4GB of main memory. The Rocks Cluster Distribution is installed on all nodes.

## Conclusion

In this article, a method for detecting epistatic multi-locus interactions in case-control data is presented. The study focuses on pure epistasis [2], which cannot be detected via single-locus analysis [42]. To overcome this difficulty, the proposed method performs an omnibus permutation test [54] on ensembles of two-locus analyses and is thus referred to as 2LOmb. The detection performance of 2LOmb is evaluated using both simulated and real data. From the simulation, 2LOmb produces a low false-positive error when the tests on null data of no association are performed. Furthermore, 2LOmb can identify all causative SNPs and outperforms a simple exhaustive two-locus analysis technique, a set association approach (SAA) [11], a correlation-based feature selection (CFS) technique [14] and a tuned ReliefF (TuRF) technique [16] in various interaction scenarios with marginal two-locus effects. These scenarios are set up by varying the number of causative SNPs, the number of SNPs in data, the sample size and the heritability. ANOVA reveals that the number of SNPs in data and the sample size influence the number of erroneous SNPs appended to the correctly-identified causative SNPs in the 2LOmb output. In contrast, the results from 2LOmb appear to be insensitive to the variation in heritability. After subjecting the data sets containing only SNPs that are screened by 2LOmb to multifactor dimensionality reduction (MDR) analysis [19], all erroneous SNPs are successfully removed. In addition, an insight into the MDR models is provided. 2LOmb is subsequently applied to a real case-control type 2 diabetes mellitus (T2D) data set, which is collected from a UK population by the Wellcome Trust Case Control Consortium (WTCCC) [3]. The original genome-wide data set is first reduced by selecting only SNPs that locate within or near 372 candidate genes reported by the Human Genome Epidemiology Network (HuGENet) [62]. In addition, the selected SNPs must exhibit no marginal single-locus effects. The final data set, which consists of 1,999 case samples and 3,004 control samples, contains 7,065 SNPs from 370 candidate genes. 2LOmb identifies 11 intronic SNPs that are associated with the disease. These SNPs are located in *PGM1*, *LMX1A*, *PARK2 *and *GYS2*. The 2LOmb result suggests that there is no interaction between each pair of the identified genes that can be described by purely epistatic two-locus interaction models. Moreover, there are no interactions between these four genes that can be described by purely epistatic multi-locus interaction models with marginal two-locus effects. This evidence of genetic association for these four genes leads to an alternative explanation for the aetiology of T2D in the UK population. It also implies that SNPs from genome-wide data which are usually discarded after single-locus analysis confirms the null hypothesis of no association can still be useful for genetic association studies of complex diseases.

## Methods

### Pure epistasis model

The pure epistasis model of interest is proposed by Culverhouse et al. [42]. The model describes a restriction or constraint for penetrance of each genotype constituting the interaction model. Consider a two-locus model that captures an interaction between loci A and B, let *A *and *a *be the major (common) and minor (rare) alleles at locus A. Similarly, let *B *and *b *be the major and minor alleles at locus B. At each locus, the genotype is represented by characters 0, 1 or 2 where 0 denotes a homozygous wild-type genotype (*AA *and *BB*), 1 denotes a heterozygous genotype (*Aa *and *Bb*) and 2 denotes a homozygous variant or homozygous mutant genotype (*aa *and *bb*). *f*_*ij *_∈ [0, 1] is defined as the disease penetrance of the two-locus genotype *ij *that consists of genotype *i *at locus A and genotype *j *at locus B. The marginal penetrances *M*_*Ai *_for genotype *i *at locus A and *M*_*Bj *_for genotype *j *at locus B are given by

(4)

and

(5)

where *p*_*A *_and *p*_*B *_are the major allele frequencies. Equations 4 and 5 are usually represented by a penetrance table as illustrated in Table 13. The two-locus interaction model is a pure epistasis model if

**Table 13:** Penetrances for a two-locus interaction model.

	**Penetrance of Genotype**			
**Genotype**	***BB***	***Bb***	***bb***	**Marginal Penetrance**
*AA*	*f*_00_	*f*_01_	*f*_02_	*M*_*A*0_
*Aa*	*f*_10_	*f*_11_	*f*_12_	*M*_*A*1_
*aa*	*f*_20_	*f*_21_	*f*_22_	*M*_*A*2_
Marginal Penetrance	*M*_*B*0_	*M*_*B*1_	*M*_*B*2_	*K*

*f*_*ij *_is the disease penetrance of genotype *ij*. *M*_*Ai *_and *M*_*Bj *_are the marginal penetrances for genotype *i *at locus A and genotype *j *at locus B, respectively. *M*_*Ai *_= *M*_*Bj *_= *K*, ∀*i*, *j *∈ {0, 1, 2} in a pure epistasis model.

(6)

where *K *is the disease prevalence. Obviously, many combinations of penetrance *f*_*ij *_satisfy the condition given in equation 6. Culverhouse et al. [42] suggest that a pure epistasis model with the maximum heritability is particularly useful in association studies. The heritability (*h*^2^) of the two-locus interaction model is defined by

(7)

where *V*_*T *_= *K*(1 - *K*) is the total variance of the dichotomous phenotypes in the population and *V*_*I *_is the epistatic variation attributable to the genotypes. *V*_*I *_is defined by

(8)

The search for feasible penetrance *f*_*ij *_that also maximises the heritability or other variance-based objectives can be treated as a constraint optimisation problem. Many algorithms including a double description method [42] and a genetic algorithm [83] have been proven to be suitable for the task.

Culverhouse et al. [42] have identified the maximum heritability of purely epistatic two-locus and multi-locus interaction models for various values of disease prevalence. For instance, the maximum heritability of a two-locus interaction model for *p*_*A *_= *p*_*B *_= 0.5 with the penetrances in Table 14 is

**Table 14:** Two-locus penetrances that lead to the maximum heritability  (*K*) = 2*K*/(1 - *K*) for *K *∈ (0, 1/4].

	**Penetrance of Genotype**		
**Genotype**	***BB***	***Bb***	***bb***
*AA*	0	0	4*K*
*Aa*	0	2*K*	0
*aa*	4*K*	0	0

All allele frequencies are equal (*p*_*A *_= *p*_*B *_= 0.5).

(9)

When a two-locus interaction model is expanded into a multi-locus interaction model, the marginal penetrance equality constraint must be extended to cover all loci. Furthermore, the expression for *V*_*I *_must also be expanded to cover additional genotypes while the expression for *V*_*T *_remains unchanged. With the necessary model expansion, the maximum heritability of a three-locus interaction model for *p*_*A *_= *p*_*B *_= *p*_*C *_= 0.5 with the penetrances in Table 15 is given by

**Table 15:** Three-locus penetrances that lead to the maximum heritability  (*K*) = 9*K*/(1 - *K*) for *K *∈ (0, 1/16].

	**Penetrance of Genotype**								
**Genotype**	***CC***			***Cc***			***cc***		
	***BB***	***Bb***	***bb***	***BB***	***Bb***	***bb***	***BB***	***Bb***	***bb***
*AA*	0	0	16*K*	0	0	0	0	0	0
*Aa*	0	0	0	0	4*K*	0	0	0	0
*aa*	0	0	0	0	0	0	16*K*	0	0

All allele frequencies are equal (*p*_*A *_= *p*_*B *_= *p*_*C *_= 0.5).

(10)

Similarly, the maximum heritability of a four-locus interaction model for *p*_*A *_= *p*_*B *_= *p*_*C *_= *p*_*D *_= 0.5 with the penetrances in Table 16 is

**Table 16:** Four-locus penetrances that lead to the maximum heritability  (*K*) = 35*K*/(1 - *K*) for *K *∈ (0, 1/64].

	**Penetrance of Genotype**									
**Genotype**		***CC***			***Cc***			***cc***		
		***DD***	***Dd***	***dd***	***DD***	***Dd***	***dd***	***DD***	***Dd***	***Dd***
	*BB*	0	0	0	0	0	0	0	0	0
*AA*	*Bb*	0	0	0	0	0	0	0	0	0
	*bb*	0	0	64*K*	0	0	0	0	0	0
	*BB*	0	0	0	0	0	0	0	0	0
*Aa*	*Bb*	0	0	0	0	8*K*	0	0	0	0
	*bb*	0	0	0	0	0	0	0	0	0
	*BB*	0	0	0	0	0	0	64*K*	0	0
*aa*	*Bb*	0	0	0	0	0	0	0	0	0
	*bb*	0	0	0	0	0	0	0	0	0

All allele frequencies are equal (*p*_*A *_= *p*_*B *_= *p*_*C *_= *p*_*D *_= 0.5).

(11)

Additional details about the maximum heritability and the corresponding two-locus and multi-locus penetrance tables for other values of disease prevalence can be found in Culverhouse et al. [42]. In this article, the simulated data sets are generated to achieve the maximum heritability of 0.01, 0.025 and 0.05. The values of disease prevalence that lead to the target heritability for two-, three- and four-locus interaction models are given in Table 17.

**Table 17:** Disease prevalence that gives the target maximum heritability of 0.01, 0.025 and 0.05 for two-, three- and four-locus interaction models.

	**Prevalence (*K*)**		
**Model**	(*K*) = 0.01	(*K*) = 0.025	(*K*) = 0.05
Two-locus	0.004975	0.012346	0.024390
Three-locus	0.001110	0.002770	0.005525
Four-locus	0.000286	0.000714	0.001427

### genomeSIM

genomeSIM is a simulation package for generating case-control samples in large-scale and genome-wide association studies [60]. The package is capable of producing many realistic scenarios, which can be observed in a population and genetic samples, including linkage disequilibrium, phenocopy and genotyping errors. The case/control status of each sample is determined from the penetrance-based genetic models or interaction models. As a result, the package can accommodate many epistasis models including the one proposed by Culverhouse et al. [42]. A data set can be produced via two modes: a population-based simulation and a probability-based simulation. In the population-based simulation, an initial population is generated according to the predefined allele frequency of each SNP. Then further generations are created by crossing the genotype strings within successive generations until the specified number of generations is reached. The resulting data set contains a population-dependent case and control samples that follow a forward-time simulation strategy. In contrast, genotype strings are incrementally generated without any string crossing for only one generation in the probability-based simulation. The creation of new strings is terminated only when the desired numbers of case and control samples are obtained. In this study, the probability-based simulation is used to produce all case and control samples where the simulation parameter setting is given in the supplement (see Additional file 2). genomeSIM is available upon request to Scott M. Dudek at the Vanderbilt University dudek@chgr.mc.vanderbilt.edu.

### Set association approach

A set association approach (SAA) is an association detection technique based on an omnibus permutation test on sets of candidate SNPs [11]. The test captures information about genotyping errors, deviation from Hardy-Weinberg equilibrium (HWE) and allelic association. In the first step, the genotype distribution for each SNP in the control samples is checked for HWE. Then, the number of SNPs that is to be excluded from the study (*n*_*d*_) is set to the number of SNPs in the control samples that deviate from HWE. Two test statistics are subsequently calculated for each SNP: an allelic association statistic and a statistic for the deviation from HWE of each SNP in the case samples. The allelic association statistic is a *χ*^2 ^statistic which is calculated from the contingency table of alleles or genotypes with disease status. On the other hand, a *χ*^2 ^statistic for the deviation from HWE of each SNP in the case samples indicates the level of association. A large deviation from the equilibrium usually signifies strong association between a SNP and the disease. However, an excessively large deviation may be the result of genotyping errors. *n*_*d *_SNPs with largest test statistics for the deviation from HWE are hence excluded from the consideration.

The test statistics for the allelic association and deviation from HWE are multiplied together to form a single *S *statistic for each remaining SNP. SNPs are then ranked according to their *S *statistics. A preset number of SNPs with highest ranks are considered for association. The first candidate SNP set contains only the SNP with the highest rank (the highest *S *statistic). The *p*-value for this first set is determined from a permutation simulation where the case and control labels are randomly permuted while the numbers of case and control samples remain unchanged. In each permutation replicate, a new genotype contingency table is constructed and a new *S *statistic is subsequently obtained. The *p*-value is given by the fraction of permutation replicates with an *S *statistic greater than or equal to the *S *statistic from the original data. The second candidate SNP set consists of the first two SNPs in the rank list. The test statistic for this SNP set is the sum of *S *statistics from both SNPs. The *p*-value for the second candidate SNP set is also obtained through the permutation simulation. By progressively adding the remaining SNP with the highest rank to the previously considered candidate set and performing the permutation simulation, *p*-values for all candidate SNP sets are estimated. The sizes of candidate SNP sets have the range of one to the preset number. Among all candidate sets, the SNP set that best describes genetic association has the lowest *p*-value.

Since multiple hypotheses are postulated during the construction of candidate SNP sets, the global *p*-value for the selected candidate set must be evaluated. This is achieved through a permutation simulation in which the current raw *p*-value for the chosen candidate set is now used as the test statistic. The existing permutation replicates, created for the early estimation of the raw *p*-value, can be reused and a nested permutation simulation is hence avoided. In this study, the maximum allowable size of the candidate SNP set is the total number of available SNPs while the number of permutation replicates for *p*-value evaluation is set to 10,000. The allelic association statistic employed in the study is the *χ*^2 ^statistic that is obtained through the contingency table of genotypes with disease status. A PASCAL program for the set association approach can be obtained from the website for *S *statistic in gene mapping [84].

### Correlation-based feature selection technique

A correlation-based feature selection (CFS) technique [14] is an attribute (SNP) subset evaluation heuristic that considers both the usefulness of individual features (SNPs) in the (case-control) classification task and the level of inter-correlation among features. Each attribute subset is assigned a score given by

(12)

where *Merit*_*F *_is the heuristic merit of an *n*_*c*_-attribute subset *F*, _*cf *_is the average feature-class correlation and _*ff *_is the average feature-feature inter-correlation. An attribute subset receives a high merit score if it contains features that are highly correlated with the class and at the same time have low inter-correlation among one another. An application of a best-first search for the best subset identification is carried out to avoid searching through all possible attribute subsets. CFS has been integrated into a Weka package [85,86].

### Tuned ReliefF

A tuned ReliefF (TuRF) algorithm is a ranking algorithm for identifying genetic markers which are important in case-control classification [16]. TuRF is built on a ReliefF engine [15]. ReliefF randomly picks a sample from the (case-control) data and identifies its *n*_*k *_nearest neighbours from the same class and another *n*_*k *_nearest neighbours from the opposite class. The attribute values--the genotypes in this application--of the neighbour samples are compared to that of the randomly picked sample and are subsequently used to update the relevance score for each attribute (genetic marker). This process is repeated for a specified number of samples, which is limited by the total sample size. The rationale of ReliefF is that an attribute which is important for the classification should have different values for samples from different classes and have the same value for samples from the same class. The relevance score of an attribute have a range from -1 (not relevant) to +1 (highly relevant). TuRF exploits the capability of ReliefF by repeatedly executing ReliefF and removing a portion of worst attributes at the end of each execution. This leads to the reevaluation of remaining attributes and, hence, reduces the effects of attribute noise on the attribute screening. In this study, the number of repetitions for random sample picking in the ReliefF part is equal to the total number of case-control samples while the neighbourhood size (*n*_*k*_) for the relevance score calculation is set to ten. Furthermore, the worst 1% of SNPs is removed at the end of each ReliefF iteration (TuRF 1%). TuRF has been integrated into the current distribution of multifactor dimensionality reduction (MDR) software.

### Multifactor dimensionality reduction

**Figure 13:**
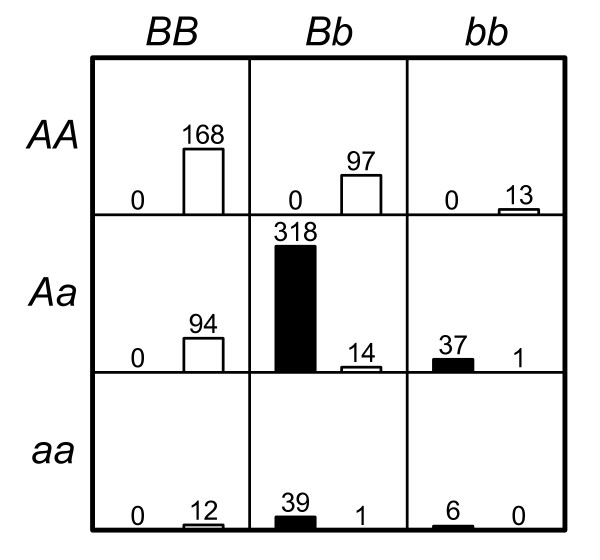
**An MDR decision table that is constructed using a balanced case-control data set with the sample size of 800**. The genotype of each sample is determined from two SNPs. The table consists of nine cells where each cell represents a unique genotype. The left (black) bar in each cell represents the number of case samples while the right (white) bar represents the number of control samples. The cells with genotypes *AABB*, *AABb*, *AAbb*, *AaBB *and *aaBB *are labelled as protective genotypes while the cells with genotypes *AaBb*, *Aabb*, *aaBb *and *aabb *are labelled as disease-predisposing genotypes.

A multifactor dimensionality reduction (MDR) method is a wrapper-based technique that is capable of identifying the best genetic marker combination among possible markers for the separation between case and control samples [19]. Similar to other wrapper-based methods, an *n*_*f *_-fold cross-validation technique provides a means to determine the prediction accuracy of the candidate marker model. Basically, the combined case and control samples are randomly divided into *n*_*f *_folds where *n*_*f *_- 1 folds of samples are used to construct a decision table while the remaining fold of samples is used to identify the prediction capability of the constructed decision table. The decision table construction and testing procedure is repeated *n*_*f *_times. Hence, the samples in each fold are always used both to construct and to test the decision table. The number of cells in a decision table is given by  where *n*_*c *_is the number of candidate markers selected from possible markers and *G *is the number of possible genotypes according to the marker. For a SNP, which is a bi-allelic marker, *G *is equal to three. During the decision table construction, each cell in the table is filled with case and control samples that have their genotype corresponds to the cell label. The ratio between numbers of case and control samples provides the decision for each cell whether the corresponding genotype is a protective or disease-predisposing genotype. An example of decision table construction is illustrated in Figure 13.

The prediction accuracy of the decision table is subsequently evaluated by counting the numbers of case and control samples in the testing fold that their disease status can correctly be identified using the constructed decision rules. The process of decision table construction and evaluation must be cycled through all or some of possible  - 1 combinations where *n*_*m *_is the total number of available markers in the study. The best genetic marker combination is determined from two criteria: prediction accuracy and cross-validation consistency. Each time that a testing fold is used for the prediction accuracy determination, the accuracy of the interesting marker combination model is compared with that from other models that also contain the same number of markers. The model that consistently ranks the first in comparison to other choices with the same number of markers has high cross-validation consistency. Prediction accuracy is the main criterion for decision making while cross-validation consistency is only used as an auxiliary measure. Cross-validation consistency generally confirms that the high rank model can consistently be identified regardless of how the samples are divided for cross-validation. In a situation where two or more models with different number of markers are equally good in terms of prediction accuracy and cross-validation consistency, the most parsimonious model--the combination with the least number of markers--is chosen as the best model.

After the best model has been selected, a permutation test is used to assess the probability of obtaining prediction accuracy that is at least as large as or larger than that observed in the original data from randomised data. This represents the probability that the null hypothesis of no association is true. Each permutation replicate is constructed by randomly assigning the case/control status to each sample with the numbers of case and control samples remaining fixed. MDR analysis is subsequently carried out to obtain the prediction accuracy of each permutation replicate. The empirical *p*-value is denoted by the fraction of permutation replicates with the prediction accuracy greater than or equal to the prediction accuracy obtained from the original data. MDR software, which incorporates many additional features including interaction visualisation via dendrograms and genetic marker screening via a *χ*^2 ^test, an odds ratio test, ReliefF and TuRF, is available from its homepage [87].

### JLIN

JLIN or a Java LINkage disequilibrium plotter is a computer program for visualisation of linkage disequilibrium analysis [63]. The program is capable of displaying many statistical measures including *D' *[64] and *r*^2 ^[65]. The program is publicly available from the Centre for Genetic Epidemiology and Biostatistics, University of Western Australia [88].

### Interaction dendrogram

An interaction dendrogram is a graphical tool for the visualisation of relationships among attributes (SNPs) [68,69]. The interaction dendrogram is constructed via hierarchical clustering analysis and is embedded into MDR software [87]. The dendrogram illustrates the entropy-based interaction between attributes by displaying interacting or related attributes closely together as adjacent leaves in a tree. At the same time, independent attributes are placed far apart from one another. In addition, the conclusion regarding whether the interaction between attributes is synergistic or redundancy is present can be deduced.

## Availability and requirements

The 2LOmb program for Windows platforms and examples of simulated data are available at .

## List of abbreviations

2LOmb: omnibus permutation test on ensembles of two-locus analyses; ALT: alanine transaminase; ANOVA: analysis of variance; AST: aspartate transaminase; CFS: correlation-based feature selection; CI: confidence interval; CVC: cross-validation consistency; *DIO2*: deiodinase, iodothyronine, type II; E2LA: exhaustive two-locus analysis; *EGFR*: epidermal growth factor receptor (erythroblastic leukemia viral (v-erb-b) oncogene homolog, avian); FAMHAP: software for single-marker analysis and joint analysis of unphased genotype data from tightly linked markers (haplotype analysis); FUSION: Finland-United States Investigation of NIDDM Genetics; genomeSIM: simulation package for generating case-control samples in large-scale and genome-wide association studies; *GYS2*: glycogen synthase 2 (liver); *HNF4A*: hepatocyte nuclear factor 4, alpha; HuGENet: Human Genome Epidemiology Network; HWE: Hardy-Weinberg equilibrium; JLIN: Java LINkage disequilibrium plotter; *KCNJ11*: potassium inwardly-rectifying channel, subfamily J, member 11; LD: linkage disequilibrium; LIM domains: protein structural domains that are named after their initial discovery in the proteins Lin11, Isl-1 and Mec-3; *LMX1A*: LIM homeobox transcription factor 1, alpha; MDR: multifactor dimensionality reduction; NIDDM: noninsulin-dependent diabetes mellitus; *PARK2*: Parkinson disease (autosomal recessive, juvenile) 2, parkin; *PGM1*: phosphoglucomutase 1; *PPARG*: peroxisome proliferator-activated receptor gamma; *RXRG*: retinoid X receptor, gamma; SAA: set association approach; SNP: single nucleotide polymorphism; T2D: type 2 diabetes mellitus; TuRF: tuned ReliefF; *UCP2*: uncoupling protein 2 (mitochondrial, proton carrier); Weka: Waikato environment for knowledge analysis; WTCCC: Wellcome Trust Case Control Consortium.

## Authors' contributions

WW conducted the literature survey, formulated the research question, implemented the proposed algorithm, designed the experiment, and collected and interpreted the computational results. AA conducted the literature survey, formulated the research question, designed the experiment and secured the access to the genomeSIM package. TP performed the statistical analysis and interpreted the statistical results. SS monitored and oversaw the execution of computer programs on the Beowulf cluster. CL provided additional comments about the genetic association study of T2D. NC conducted the literature survey, formulated the research question, designed the proposed algorithm, designed the experiment, secured the access to the T2D data from WTCCC, selected the candidate genes for the T2D association study, discussed all results, drew the conclusions and wrote the manuscript. All authors read and approved the final manuscript.

## Authors' information

WW is a Ph.D. student at the Department of Electrical Engineering, Faculty of Engineering, King Mongkut's University of Technology North Bangkok. He also received his B.Eng. and M.Eng. degrees in electrical engineering from King Mongkut's University of Technology North Bangkok. His current research interests include machine learning, evolutionary computation and bioinformatics.

AA is a Ph.D. student at the Department of Immunology, Faculty of Medicine Siriraj Hospital, Mahidol University. He also received his B.Sc. degree in pharmacy from Mahidol University. His current research interests include human genetics, genetic epidemiology, population genetics and bioinformatics.

TP is a Ph.D. student at the Department of Electrical Engineering, Faculty of Engineering, King Mongkut's University of Technology North Bangkok. He also received his B.Eng. and M.Eng. degrees in production engineering from King Mongkut's University of Technology North Bangkok. His current research interests include evolutionary multi-objective optimisation and machine learning.

SS is a part-time research assistant at the Department of Electrical Engineering, Faculty of Engineering, King Mongkut's University of Technology North Bangkok. He received his B.Eng. and M.Eng. degrees in electrical engineering from Thammasat University and King Mongkut's University of Technology North Bangkok, respectively. His current research interests include machine learning and genetic epidemiology.

CL is the Head of Division of Molecular Genetics at the Department of Research and Development, Faculty of Medicine Siriraj Hospital, Mahidol University. He also received his M.D. degree from Mahidol University. His current research interests include human genetics and genetic diseases.

NC is an associate professor of electrical engineering at King Mongkut's University of Technology North Bangkok and an adjunct professor of genetic epidemiology at Mahidol University. He received his B.Eng. and Ph.D. degrees from the Department of Automatic Control and Systems Engineering, University of Sheffield. His current research interests include evolutionary computation, machine learning and genetic epidemiology.

## Supplementary Material

Additional file 1 **List of SNPs for the association study of T2D**. This Excel spreadsheet file contains the information about 7,065 SNPs which are explored during the genetic association study of T2D. Bonferroni-corrected and uncorrected *χ*^2^'s *p*-values from single-locus analyses are also provided. Click here for file

Additional file 2 **genomeSIM parameters**. This text file contains an example of parameter setting in the genomeSIM simulation package. Click here for file
